# Accelerators, Gantries, Magnets and Imaging Systems for Particle Beam Therapy: Recent Status and Prospects for Improvement

**DOI:** 10.3389/fonc.2021.737837

**Published:** 2022-02-15

**Authors:** Edward W. Collings, Lanchun Lu, Nilendu Gupta, Mike D. Sumption

**Affiliations:** ^1^ Department of Materials Science and Engineering, College of Engineering, The Ohio State University, Columbus, OH, United States; ^2^ Department of Radiation Oncology, The James Cancer Hospital and Solove Research Institute, Wexner Medical Center and College of Medicine at the Ohio State University, Columbus, OH, United States

**Keywords:** particle beam therapy, protons, carbon ions, particle acceleration, cyclotron, image-guided particle beams, synchrotron

## Abstract

The paper begins by emphasizing the clinical and commercial importance of proton or other charged particle such as carbon ion therapy, refers to the manufacturers of such systems of which more than 120 are installed or under construction worldwide by April 2021. A general review of charged particle therapy systems refers to six manufacturers and provides in tabular form some details of systems installed in the US, Europe, Asia, and elsewhere. In a description of the principles of particle beam therapy a comparison is made of the properties of photons (x-rays) versus protons and protons versus carbon ions. A brief discussion of accelerators in general is followed by descriptions of cyclotrons (including the isosynchronous cyclotron and the synchrocyclotron) and synchrotrons. An interesting case study describes the evolution of a normal-conducting 220 ton cyclotron into an iron-free synchrocyclotron weighing only 5 tons. The general principles of beam handling and gantry design are described. Subsequent sections describe gantry magnets in detail - normal conducting gantry magnets, superconducting gantry magnets for proton- and carbon therapy. Mention is made of a novel CERN-designed superconducting toroidal gantry for hadron therapy, GaToroid. This device, operating under steady state current and magnetic field, is able to deliver a beam at discrete angles over a range of treatment energies. Also considered are low temperature superconducting (LTS) and high temperature superconducting (HTS) magnet windings, and the choice of REBCO conductors for cryogen-free carbon-ion gantries. Finally, the paper mentions an important “Prospect for Improvement”, viz: the introduction of MRI image guidance. A well-known property of the particle beam as it passes through tissue is its energy dependent absorption that rises to a pronounced peak (the Bragg peak) at the end of its range. In order to take advantage of this effect the exact targeting of the tumor and positioning of the patient should be guided by imaging visualization using X-ray, CT, and hopefully advanced MRI. Unlike MRI-guided photon therapy the direct interaction of the magnetic field with the charged particle beam presents a huge challenge such that MRI image-guided proton/particle therapy has not yet been available in clinical practice. Modeling studies have been undertaken on the general topic of beam-line/magnetic field interaction using, for example, the software GEANT4 (GEometry And Tracking) a platform for simulating the passage of charged particles through matter using a Monte Carlo method.

## 1 Introduction: Clinical Status, Market Report and Predictions, Facilities

### 1.1 Clinical Status 2014-2030

2014: During year 2014 more than 140 treatment rooms were serving 14,500 patients

2015: In year 2015 only 0.5% of radiation-needy patients were treated with proton therapy.

2019: By year 2019 330 patient treatment rooms are expected to be available, but even then only 1% of radiation-needy patients will be able to receive particle therapy.

2030: By year 2030 it is expected that 1200 to 1800 treatment rooms will be open to patients worldwide. But even 1800 rooms will allow only 5% of radiation-needy patients to receive particle therapy.

### 1.2 Market Report and Predictions 

2000: In year 2000 the proton therapy market was valued at US$ 1 million

2015: During year 2015 the market reached US$ 800 million (1, 2)

2019: In year 2019 the market is expected to reach more than US$ 1 billion

During 2000-2014 the average annual growth rate was almost 15% p.a.

During 2010-2014 the growth rate underwent acceleration to 22% p.a.

2030: By year 2030 the proton therapy world market is expected to be worth US$ 3.5 to 6.6 billion in response to an annual growth rate of 11% to 16%.

### 1.3 Facilities

In 2015 there was reported to be 15 manufacturers or developers of ion therapy equipment, and it was predicted that by 2019 the market would be dominated by 5 of those companies. Important manufacturers include: Ion Beam Applications (IBA), Varian, Sumitomo, Hitachi, Toshiba, Siemens, and Mitsubishi, the latter having installed at least 11 proton- or carbon-ion centers in Japan. Of the more than 60 proton therapy facilities worldwide 25 are located in the US. After a slow start the proton therapy market began to accelerate in response to: (i) a growing recognition of the effectiveness of particle therapy, (ii) the recent introduction of lower cost compact systems and single-treatment-room centers. CERN, with its experience in proton accelerator technology (e.g. the LHC) continues to make important contributions to ion therapy. Proton/carbon-ion centers in Japan, Europe-and-Elsewhere, and the U.S. are listed in **Tables 1**–**3**, respectively.

**Table 1:** Partial listing of particle therapy centers in europe and elsewhere.

Country	City	Institution	First patient
China	Zibo	Wanje Proton Therapy Center	2004
China	Lanzhou	Lanzhou Heavy Ion Therapy Research Center, Institute of Modern Physics, CAS	2006
China	Shanghai	Shanghai Proton and Heavy Ion Center	–
Taiwan	Taipei	Chang Gung Memorial Hospital (CGMH)	2012
Czech Republic	Prague	Proton Therapy Center Czech	2012
France	Nice	Centre Laccassagne	1991
France	Caen	Centre National de Radiotherapy	–
France	Orsay	Centre Protontherapy de l’Institut Curie	–
Germany	Berlin	HMI	1998
Germany	Heidelberg	Heidelberg Ion Therapy Center	2009
Germany	Munich	Rinecker	2009
Germany	Dresden	Universitätsklinikum Carl Gustav Carus	2014
Germany	Essen	Westdeutsches Protonentherapiezentrum Essen	2013
Germany	Kiel	University Schleswig-Holstein (UC S-H)	na
Germany	Marburg	Rhön-Klinikum	na
Italy	Pavia	CNAO Pavia	2009
Italy	Trento	Agenzia Provinciale Per la Protonterapia (AtreP)	2012
Italy	Catania	Laboratori Nazionali del Sud	
Korea	Seoul	Samsung Hospital	2014
Korea	Ilsan	Korean National Cancer Center	2007
Netherlands	Groningen	University Medical Center Groningen (UMCG)	–
Poland	Krakow	Instytut Fizyki Jadrowej, Polish Acad. Sci.	2013
Russia	Dimitrovgrad	Federal High-Tech Medical Center	2013
Russia	St Petersburg	Center of Nuclear Medicine	2016
Russia	Moscow	Institute for Theoretical and Experimental Physics	–
Russia	Dubna	Joint Institute for Nuclear Research	–
Sweden	Uppsala	Skandion Kliniken	2013
Switzerland	Villigen	Paul Scherrer Institut	1984
Saudi Arabia	Riyadth	King Fahd Medical City	2015
South Africa	Somerset West	iThemba Labs	1993
United Kingdom	Newport	The Rutherford Cancer Center South Wales	–
United Kingdom	Clatterbridge	The Clatterbridge Cancer Center	–

**Table 2:** Particle therapy centers in Japan.

Facility	Location	Ion Species
Aizawa Proton Therapy Center (PTC)	Nagano Prefecture, Matsumoto	Proton
Fukui Prefectural Hospital Proton Therapy Center	Yotsui, Fukui City	Proton
Gunma University Heavy Ion Medical Center	Maebashi, Gunma	Carbon
Hokaido University Hospital	Sapporo-shi, Hokaido	Proton
Hyogo Ion Beam Medical Center	Tatsuno-shi, Hyogo	Proton Carbon
Medipolis International Proton Beam Treatment Center	Ibusuki-shi, Kagoshima Prefecture	Proton
National Cancer Center Hospital East	Kashiwa-shi, Chiba	Proton
National Institute of Radiological Sciences	Inage-ku, Chiba-shi, Chiba	Carbon Heavy ion
Nagoya Proton Therapy Center	Kita-ku, Nagoya	Proton
Proton Medical Research Center, University of Tsukuba	Ibaraki Prefecture, Tsukuba	Proton
Saga Heavy Ion Cancer Treatment Center	Tosu-shi, Saga Prefecture	Carbon
Shizuoka Cancer Center	Sunto-gun, Shizuoka Prefecture	Proton
Southern Tohoku PTC	Koriyama, Fukushima	Proton
Tsuyama Chuo Hospital Proton Beam Cancer Center	Okayama	Proton
Heavy Ion Medical Accelerator in Chiba, (HIMAC)	Chiba	He, Ne, C, Si, Ar

**Table 3:** Proton therapy centers in the US (3, 4).

Arizona	Mayo Clinic Proton Beam Therapy Program, Phoenix, AZ
California	Scripps Proton Therapy Center, San Diego, CA
California	James M. Slater, M.D. Proton Treatment and Research Center at Loma Linda
University	Medical Center, Loma Linda, CA
California	UCSF Ocular Tumor Proton Radiation Program, Crocker Nuclear Laboratory, San Francisco, CA
California	UC Davis Cancer Center, Lawrence Livermore Nat’l Laboratory & Tomotherapy Inc, CA
Florida	Ackerman Cancer Center, Jacksonville, FL
Florida	University of Florida Health Proton Therapy Institute, Gainesville, FL
Florida	UF Health Cancer Center at Orlando Health, Orlando, FL
Florida	Baptist Health South Florida, FL
Illinois	Northwestern Medicine Chicago Proton Center, Chicago, IL
Louisiana	Willis-Knighton Health System, Shreveport, LA
Maryland	Maryland Proton Treatment Center, Baltimore, MD
Massach’tts	Francis H. Burr Proton Center at Mass. General Hospital, Boston, MA
Michigan	Beaumont Proton Therapy Center, Beaumont Hospital, Royal Oak, MI
Minnesota	Mayo Clinic Proton Beam Therapy Program, Rochester, MI
Missouri	Barnes Jewish Hospital (Washington University) St. Louis, MO
Missouri	S. Lee Kling Proton Therapy Center at the Siteman Cancer Center, St Louis, MO
New Jersey	ProCure Proton Therapy Center in partnership with Princeton Radiation Oncology Group and CentraState Healthcare System, Somerset, NJ
New Jersey	Laurie Proton Therapy Center at Robert Wood Johnson University Hospital, New Brunswick, NJ
Ohio	Cincinnati Children’s/UC Health Proton Therapy Center, Liberty Township, OH
Ohio	University Hospital’s Seidman Cancer Center, Case Medical Center, Cleveland, OH
Oklahoma	ProCure Proton Therapy Center, at the INTEGRIS Cancer Campus, Oklahoma City, OK
Oklahoma	Stevenson Cancer Center, Oklahoma City, OK
Pennsylvania	The Roberts Proton Therapy Center at University of Pennsylvania Health System,Philadelphia, PA
Tennessee	Provision CARES Proton Therapy Center, Knoxville, TN
Tennessee	St Jude Red Frog Events Proton Therapy Center, Memphis, TN
Texas	Texas Center for Proton Therapy, Irving, TX
Texas	M.D. Anderson Cancer Center’s Proton Center, Houston, TX
Virginia	Hampton University Proton Therapy Institute, Hampton, VA
Washington	SCCA Proton Therapy Center, Seattle, WA
Washington DC	Medstar Georgetown University Hospital, Washington DC

An increasing interest is also being shown in carbon/heavy-ion therapy, institutions offering which include: The National Institute for Radiological Sciences (Chiba), Hyogo Ion Beam Medical Center (Hyogo), Gunma University Heavy Ion Medical Center (Gunma), Saga Heavy Ion Medical Accelerator in Tosu (Saga), and Shanghai Proton and Heavy Ion Center (SPHIC, Siemens). In 2019 the program “Next Ion Medical Machine Study” (NIMMS) was established to support R&D based on CERN accelerator technology relevant to heavy ion therapy. It is also interesting to note that in a partnership between IBA and Toshiba the latter will become the Japanese distributor of IBA’s Proteus ONE compact single-room proton therapy facility, and IBA will become Toshiba’s carbon-therapy agent outside Japan. Carbon-ion centers worldwide are listed in **Table 4**.

**Table 4:** Carbon-Ion centers worldwide^(a)(b).^.

Center Name	Institution	Country	City	Date of Operation
The Center for Ion Therapy and Research	MedAustron	Austria	Wiener Neustadt	2017
Heavy Ion Research Facility	Institute of Modern Physics, Chinese Academy of Science	China	Lanzhou	2006
Shanghai Proton and Heavy Ion Center	Fudan University Shanghai Cancer Center	China	Shanghai	2014
Heidelberg Ion-Beam Therapy Center	University of Heidelberg	Germany	Heidelberg	2009
Marburger Ionenstrahl-Therapiezentrum	Heidelburg University Hospital and University Hospital Giessen and Marburg	Germany	Marburg	2015
Foundation CNAO	National Centre for Oncological Treatment CNAO	Italy	Pavia (Milan)	2011
Heavy Ion Medical Accelerator in Chiba	Japanese National Institute of Radiological Sciences	Japan	Chiba	1994
Gunma University Heavy Ion Medical Center	Gunma University Heavy Ion	Japan	Gunma	2012
Hyogo Ion Beam Medical Center	Medical Excellence JAPAN	Japan	Hyogo	2001
Kyusho International Heavy Particle Line Cancer Treatment Center		Japan	Tosu	2013
i-Rock ion-beam Radiation Oncology Center	Kanagawa Cancer Center	Japan	Yokohama	2015

^(a)^Mostly based in a list published by the Chordoma Foundation.

^(b)^See also (5).

## 2 Principles of Particle Beam Therapy

### 2.1 Reviews of Charged Particle Therapy and Systems

Review articles on proton therapy (6, 7) begin by citing the work of Robert Wilson of the Lawrence Berkeley Laboratory who in a paper published in 1946 (8) was the first to point out the importance of the proton Bragg peak for targeted radiation therapy. As protons pass through tissue at velocity *v* they continuously lose kinetic energy by inelastic Coulomb scattering against atomic electrons. The rate of energy loss, being proportional to 1/*v^2^*, rises sharply as the protons slow down, the end of their range, and form the Bragg peak. Protons also undergo repulsive non-elastic interactions with atomic nuclei and will be deflected from their original path. The product of such interactions may be secondary protons, heavier ions, neutrons, and gamma rays. These non-elastic proton-nucleus interactions, although less frequent than proton-electron ones have a stronger effect (9).

For a detailed description of the history of proton therapy systems reference (7) is recommended. That same article also gives the locations and provides technical details (as of 2010) of 6 commercial proton beam therapy systems (PTS), viz: The IBA Proteus^®^ 235 PTS, Sumitomo PTS, Varian PTS, Still River Systems Monarch 250 (Mevion Medical Systems) PTS, Hitachi PROBEAT PTS, and Mitsubishi PTS. The Hitachi and Mitsubishi systems are based on synchrotron acceleration and the others on cyclotrons. Mitsubishi is particularly active in Japan; a list of their installed proton- and carbon-ion systems is given in **Table 5**. A complete list of Japanese installations is given in **Table 2** and lists of European and US particle therapy installations are given in **Tables 1**, **3**.

**Table 5:** Mitsubishi particle therapy systems (10).

Facility	Location	Install, n year	Ion Species
National Inst. Radiological. Sciences	Chiba-shi, Chiba	1994	Heavy Ion
Hyogo Ion Beam Medical Center	Tsuno-shi, Hyogo	2001	Proton/Carbon
Shizuoka Cancer Center	Nagaizumi-cho, Shizuoka	2003	Proton
Southern Tohoku Proton Therapy Center	Koriyama-shi, Fukushima	2008	Proton
Fukui Prefectural Hospital Proton Therapy Center	Fukui-shi, Fukui	2011	Proton
Gunma University Heavy Ion Medical Center	Maebashi-shi, Gunma	2010	Heavy Ion
Mediopolis Proton Therapy & Research Center	Ibusuki-shi, Kagoshima	2011	Proton
Saga Heavy Ion Medical Accelerator in Tosu	Tosu-shi, Saga	2013	Carbon
Okayama University/Tsuyama Chuo Hospital Proton Beam Cancer Center	Tsuyama-shi, Okayama	2016	Proton
Hakuhokai Group Osaka Proton Therapy Clinic	Osaka-shi, Osaka	Under Const’n	Proton
Hyogo Prefecture Kobe Proton Therapy Center	Kobe-shi, Hyogo	Under Const’n	Proton

Excellent companions to the present document are two recently published reviews. In a paper entitled “Superconducting Magnets for Medical Accelerators” (11) S. Prestemon offers an introduction to hadron therapy and considers the challenges presented by superconducting technology. Cyclotrons and synchrotrons are reviewed, also gantries in general including TULIP (TUrning Linac for Proton Therapy) and ULICE (for Union of Light Ion Centres in Europe) which involves 20 European installations coordinated by CNAO (Pavia, Italy, see Section 4.1). L. Rossi assembled a document entitled “HITRI+ and I-FAST: Next Eu programs for SC heavy ion therapy machine” (12) which outlined the status of ion therapy in Europe and Asia. Considered were HIT (the Heidelberg Ion Beam Therapy Centre, see Section 5.5), and HIMAC (the Heavy Ion Medical Accelerator in Chiba, see Section 5.7.1). Also described was the carbon ion superconducting gantry collaboration involving CNAO (Pavia, Italy, see Section 4.1), MedAustron (Weiner, Austria, see Section 4.1), CERN, and INFN.

### 2.2 Radiation (Photons) *versus* Ions

In convention radiation (photon) therapy the absorption of x-rays or gamma rays is intensive at the surface and decreases with distance into the subject. Thus in traditional therapy a high intensity of photons must be administered using the isocentric convergence technique to allow multiple beams with the diminished intensity focus at the tumor site. As a result, much of the photon’s energy causes damage to healthy tissue.

**Figure 1:**
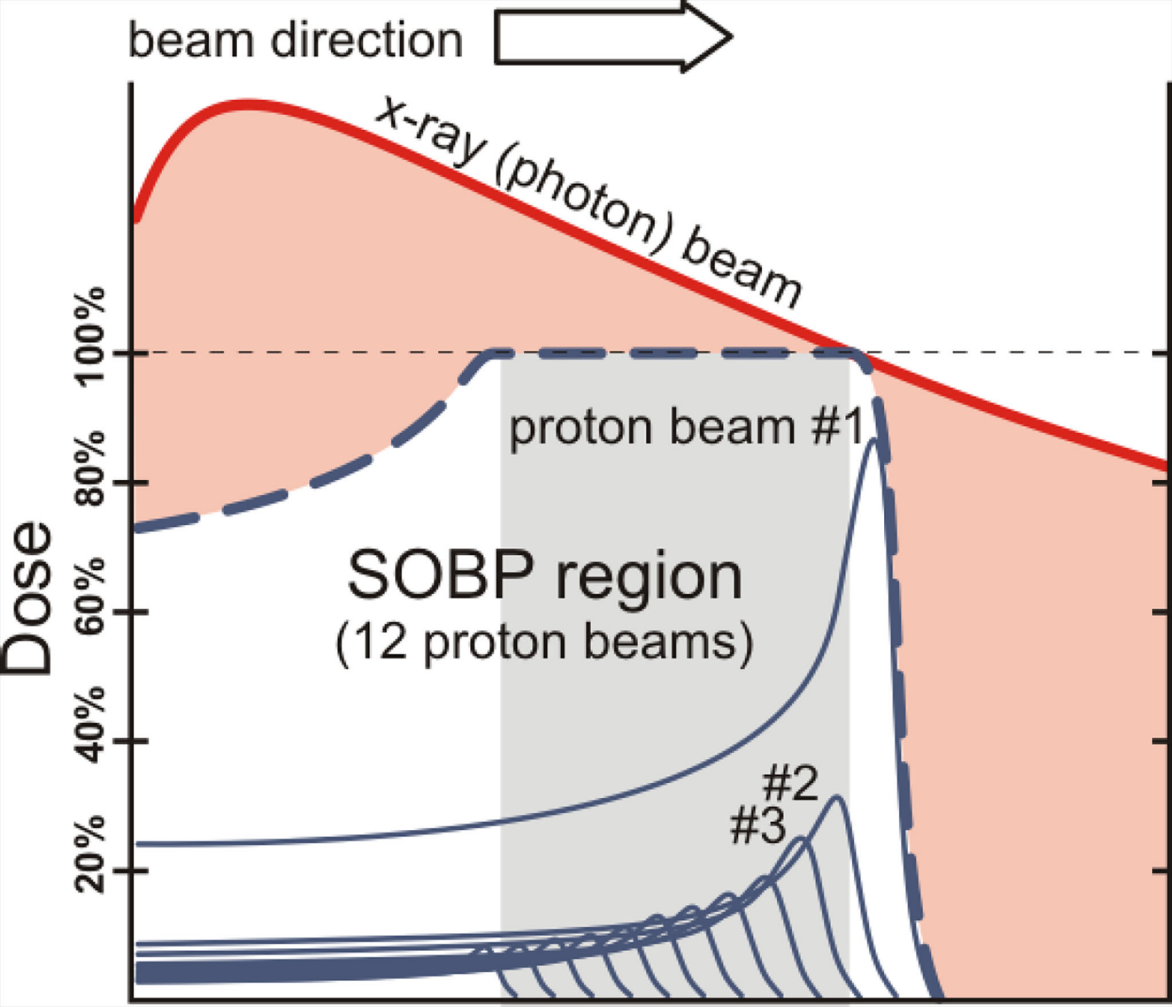
Photons versus protons showing entrance and exit doses and the spread-out Bragg peak (SOBP) – after W.P. Levin et al. (13).

**Figure 2:**
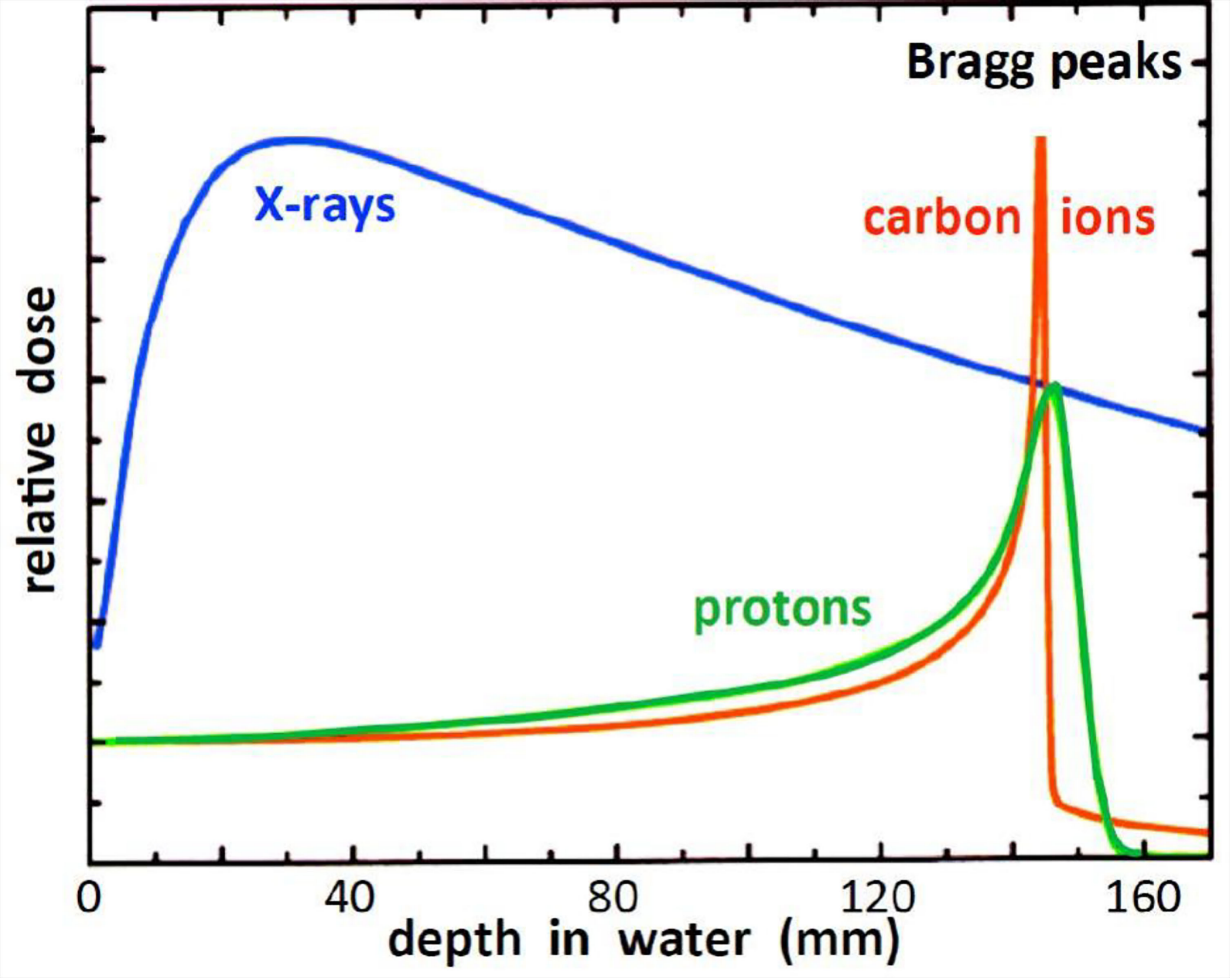
Relative dose versus depth from the body surface for photon- and charged-particle radiation – after (14).

In contrast to photons, charged particles are “silver bullets” whose interactions with matter are characterized by the Bragg curve. The energy loss by charged particle through matter is described by this curve which rises to a maximum (the Bragg peak) just before the end of the particle’s track. The peak occurs because the cross section for particle-matter interaction increases just before the particle comes to rest. In particle therapy the beam energy is adjusted either electrically (synchrotron accelerators) or by filters (cyclotron accelerators) to ensure that the Bragg peak occurs at the tumor site. **Figures 1**, **2** illustrate relative dose versus depth from the body surface for photon- and charged-particle radiation. This indicates that while photon therapy is characterized by relatively high entrance and exit doses, proton therapy has not only a lower entrance dose but a negligible exit dose. It also shows that the position of the Bragg peak can be adjusted to ensure that the tumor receives the intended radiation. This allows patients to receive high radiation doses with low risk of collateral tissue damage.

### 2.3 Protons *versus* Carbon Ions

The radiobiological effectiveness (RBE) of photon (traditional, x-ray) therapy is arguably equivalent to that of proton therapy, however as illustrated in **Figures 1** and **2**, a relatively large proportion of the photon’s energy is deposited in the entrance and exit healthy tissue regions. A distinct advantage of proton beams is that by modulating the energy to create a spread-out Bragg peak (SOBP) a large fraction of the beam energy can be deposited in the tumor site. This advantage is shared by heavy (in particular carbon) ions but in addition, being heavier than protons, they provide a higher RBE, that also increases with depth and rises to a maximum at the end of their range in the tumor region (15, 16).

Photon and proton radiation tend to produce only single-strand DNA breakage. Since cells can repair such breaks damage to both strands is required for successful treatment. Carbon ions are able to produce double-strand breaks that cannot be repaired. In other words the RBE of carbon ions against DNA is up to three times greater than that of x-rays while protons are only slightly more effective. Whereas a full treatment with protons may require 30 sessions just four days may be needed for carbon.

For hadron therapy in general, ions of He, Li, B, N, O, Ne (7), Ar, and Si (17) have also been considered, Section 4.2.

## 3 Beam Handling

### 3.1 Proton Acceleration and Handling

Beams of ionized particles are accelerated and bent by the magnetic fields of cyclotrons, synchrotrons, and dipoles. If the particle velocity, *v*, is greater than one-third *c*, the velocity of light, a relativistic correction *γ* = 1/√[1-(*v/c*)^2^] is applied to some of the equations. Thus the velocity (m/s) of an *E* MeV particle of rest mass *m_0_* can be deduced from *E* = *m_0_c* ^2^(*γ*-1) in SI units (1 MeV = 1.602x10^-13^ J). The bend radius *ρ* of a beam of particles of momentum *p* = *m_0_γv* and charge *q* deflected by a magnetic field *B* is given in SI units by:


(1) $$\rho =(p/q)/B=(m_{0}\gamma /q)/(v/B)$$

The quantity *p/q* = (*m_0_γv/q*) which has the units Tm is known as the “magnetic rigidity”, e.g (5). It represents the reluctance of the ion beam to being deflected by the field *B*. The energy-dependent rigidities of some proton and carbon beams are listed in **Table 6**, see also **Appendix A**. The table demonstrates that heavy ions beams are more difficult to deflect than proton beams and hence require stronger magnetic fields

**Table 6:** Ion energies and dose penetration depths, also calculated magnetic rigidities, fields for 1.5-m-Bend radius, and 2-T-Dipole bend radii.

Ion Species	Beam Energy, *E*, MeV/u	Beam Energy, *E*,GeV	Dose Depth, cm	Reference	Magn.Rigidity, *R*,Tm	Field for1.5 mBend Radius, *B*,T	2 T BendRadius, *ρ*,m
Proton	70			(5) p.2751	1.231	0.8	
Proton	120		10	(18) p.244	1.635	1.1	
Proton	175		20	(18) p.244	2.001	1.3	
Proton	220		30	(19) p.1	2.268	1.5	1.13
Proton	230		33	(20) p.3	2.324	1.5	1.16
Proton	250		-	(5) p.2751 (20) p.16	2.43	1.6	1.22
Carbon	400	4.80	33	(20) p.3	6.350	4.2	3.18
Carbon	425	5.10	-	(20) p.16	6.582	4.4	3.29
Carbon	430	5.16	30	(19) p.1	6.627	4.4	3.31

### 3.2 Heavy Ion (Hadron) Acceleration and Handling

Hadrons are accelerated in circular paths by cyclotrons or synchrotrons. In a modification of Equation (1) the path radius is proportional to (*M/Q*)(*v/B*) in which *M* represents ionic mass (number of protons and neutrons in the nucleus) and *Q* represents the ionic charge (number of stripped electrons). Thus as suggested in (7) a hadron synchrotron can accelerate a long list of 0.5-(*Q/M*) ions, in particular:

hydrogen (accelerated as ${\text{H}}_{2}^{+1}$ molecule). *Q/M* = 0.5
helium, *Q/M* = 2/4.0 = 0.5
lithium, *Q/M* = 3/6.9 = 0.43
boron, *Q/M* = 5/10.8 = 0.46
carbon, *Q/M* = 6/12.0 = 0.50
nitrogen, *Q/M* = 7/14.0 = 0.50
oxygen, *Q/M* = 8/16.0 = 0.50
neon, *Q/M* = 10/20 = 0.50
argon *Q/M* = 18/39.9 = 0.45
silicon Q/M = 14/28.1 = 0.50

Protons are the commonly used ions, accelerated to 220-230 MeV/u they can travel 30-33 cm into the body. Fewer systems use carbon ions which have about the same range at 400 MeV/u.

## 4 Particle Acceleration

### 4.1 Accelerators and Systems in General

For injection into a synchrotron that accelerates them to final beam energy (21) particles are typically pre-accelerated by a radiofrequency quadrupole (RFQ) cavity accelerator (7, 22) followed by a drift-tube linear accelerator (DTL) (7, 23). The numerous processes that exist for injection into cyclotrons have been discussed in detail by Mandrillon (24). Some commercial accelerators are listed in **Table 7**.

**Table 7:** Accelerators for proton and/or carbon-Ion therapy – some commercial vendors.

Accelerators*	Vendor	Notes
Cyclotrons	Varian/ACCEL Instruments	250 MeV superconducting isochronous cyclotron – see also (7)
“	IBA	Proteus^®^ 235, uses 230 MeV normal-conducting isochronous cyclotron; subsequently Proteus ONE^®^ system uses 230 MeV superconducting synchrocyclotron, S2C2
“	ProNova	ProNova SC360 superconducting isochronous cyclotron
“	Mevion Medical Systems (Still River Systems, Inc)	Mevion S250, 250 MeV superconducting synchrocyclotron with gantry-mounted 9 T cryo-cooled Nb_3_Sn-wound coils
“	Sumitomo	Normal-conducting 230 MeV isosynchronous cyclotron; cf. IBA’s first machine
Synchrotrons	Hitachi	PROBEAT-V Slow-cycling 70-250 MeV synchrotron
“	Mitsubishi	70-250 MeV synchrotrons for protons or carbon ions, 9 installed, others under construction
“	Optivus Technology	Synchrotron, 8 m diameter, continuously variable 70-250 MeV protons, cf. Loma Linda PBTS
“	Siemens	Synchrotron, 20 m diameter, 50-250 MeV/u (protons) and 85-430 MeV/u (carbon ions)
“	Toshiba	Synchrotron, 10 m diameter, 70-235 MeV

*Cyclotrons vs synchrotrons: The latter do not require energy degraders since the ions beam is accelerated to the desired energy.

CERN, with its experience in proton accelerator technology (e.g. the LHC) continues to make important contributions to ion therapy. Some 20 years ago CERN initiated the program “Proton Ion Medical Machine Study” (PIMMS) whose purpose was to produce a synchrotron tailored to treat tumors with protons and carbon ions. The design evolved into a proton and carbon ion machine built for CNAO (Italy’s National Center for Oncological Hadron Therapy, Pavia Italy, **Tables 1** and **4**). Subsequently MedAustron (Weiner, Austria, **Tables 1** and **4**) with technical support from CERN based its clinic on the CNAO design. In 2019 the program “Next Ion Medical Machine Study” (NIMMS, led by M. Vretenar) was established to support R&D based on CERN accelerator technology relevant to heavy ion therapy.

### 4.2 Synchrotrons

Therapy synchrotrons yield beams of relatively low intensity and of variable energy. Protons are injected into a ring of dipoles at 2-7 MeV and accelerated up to 70-250 MeV as the dipole field is ramped. Synchrotrons can be up to 20 m in diameter. The first hospital-installed proton therapy system was based on a Fermilab synchrotron to be further developed by Optivus Technology. Other vendors such as Hitachi, Mitsubishi, Toshiba, and Siemens followed suit, **Table 7**. But as Jongen has pointed out (7) about 75% of today’s proton therapy systems are based on cyclotron technology

### 4.3 Cyclotrons

The cyclotron consist of an RF system situated between the poles of a normal-wound or superconducting electromagnet. Considered below are the classical “traditional” cyclotron, the isochronous cyclotron, and the synchrocyclotron.

#### 4.3.1 The Conventional Cyclotron

This cyclotron incorporates a fixed-field electromagnet. The RF system consists of a pair of hollow D-shaped drift cavities excited by an oscillator of fixed frequency, *f*, given by 2π*f* = (*q/m*)*B* where *q* and *m* are the charge and mass of an assumed non-relativistic particle and *B* is the field strength. Early on a Sumitomo-ProNova collaboration produced a 230 MeV conventional normal-conducting cyclotron. In 1989 Blosser et al. (25) reported on the development at Michigan State University (MSU) of a 100 MeV superconducting cyclotron for installation at Detroit’s Harper Hospital. The cyclotron and beam delivery system are gantry mounted and will rotate through a 360° arc about the patient.

#### 4.3.2 The Isochronous Cyclotron

In this cyclotron the RF frequency is fixed but *B* varies with radius. Furthermore an azimuthal variation in *B* provides a strong focussing effect and constrains the particles in their spiral paths. The isochronous cyclotron also called the azimuthal varying field (AVF) cyclotron, is used in many of today’s systems: (i) IBA offered a resistive magnet Proteus^®^235 system which in 2010 was operating in 9 locations (7); (ii) ProNova produced a 230 MeV superconducting isochronous cyclotron which was about ½ the diameter and ¼ the weight of its resistive one mentioned above; (iii) Varian/ACCEL’s ProBeam system incorporates a 250 MeV isochronous cyclotron, the interesting feature of which is its use of four drift cavities instead of the usual two.

#### 4.3.3 The Synchrocyclotron

In the synchrocyclotron the RF frequency, *f_R_*, is decreased continuously in synchronism with the increasing velocity, *v*, of the particle within the relativistic regime. Thus *f_R_* = (*q/mγ*)*B*, where *γ* = 1/√[1-(*v/c*)^2^]. The synchrocyclotron may deploy only one D whose potential oscillates with respect to ground. The circulating particles accelerate as they drift into and out of the D. The IBA Proteus^®^235 system, a successor to their ProteusOne^®^, incorporates their model S2C2 superconducting (NbTi) synchrocyclotron and operates at a fixed 230 MeV (26). In 1989 Blosser et al. (27) reported on the design of a 250 MeV superconducting gantry-mounted synchrocyclotron system for proton therapy.

### 4.4 Evolution of Ion-Beam Therapy Accelerators

A Sumitomo-ProNova Solutions collaboration produced a normal-conducting 230 MeV cyclotron 4.4 m in diameter weighing 220 tons. The smaller ProNova SC360 system used a superconducting 230 MeV isochronous cyclotron 2.8 m in diameter weighing 50 tons. The Massachusetts Institute of Technology (MIT) in collaboration with ProNova has been working on the design and construction of an iron-free variable energy (70-230 MeV) synchrocyclotron also 2.8 m in diameter but weighing only 5 tons (28). Several advantages accrue from the use of this accelerator: (i) its variable-energy capability removes the need for graphite energy degradation, (ii) its low weight would make it very attractive for gantry mounting, (iii) gantry mounting eliminates the need for beam-directing magnets. Taken together these advantages lead to an attractive proton beam therapy system.

## 5 Components of the Ion Beam Therapy System

### 5.1 Beam Energy Adjustment

Before it enters the treatment area or gantry the ion beam requires energy adjustment. Therapy cyclotrons generate a fixed-energy beam of typically 230 MeV, the needed energy variation between 60 and 230 MeV being achieved by passing the beam through an “energy degrader, an absorber of variable thickness such as two opposite-facing wedges of graphite. These are followed by a magnetic analyzer consisting of a combination of dipole magnets and collimators (20). The “Energy Selection System” of Ion Beam Applications S.A. (IBA) allows tuning from 60-230 MeV in less than 1 second. Although the beam loss by degradation can be as much as a factor of one hundred or more typical cyclotrons deliver sufficient beam intensity to make up for this. With regard to synchrotron sources, rapid energy variation can be achieved by extracting the ion beam at different times in the acceleration cycle (20). The synchrotron can deliver proton beams in more than 90 energy steps between 73 MeV and 222 MeV corresponding to penetration depths in water of 4 to 31 cm (29).

### 5.2 The Gantry

**Figure 3:**
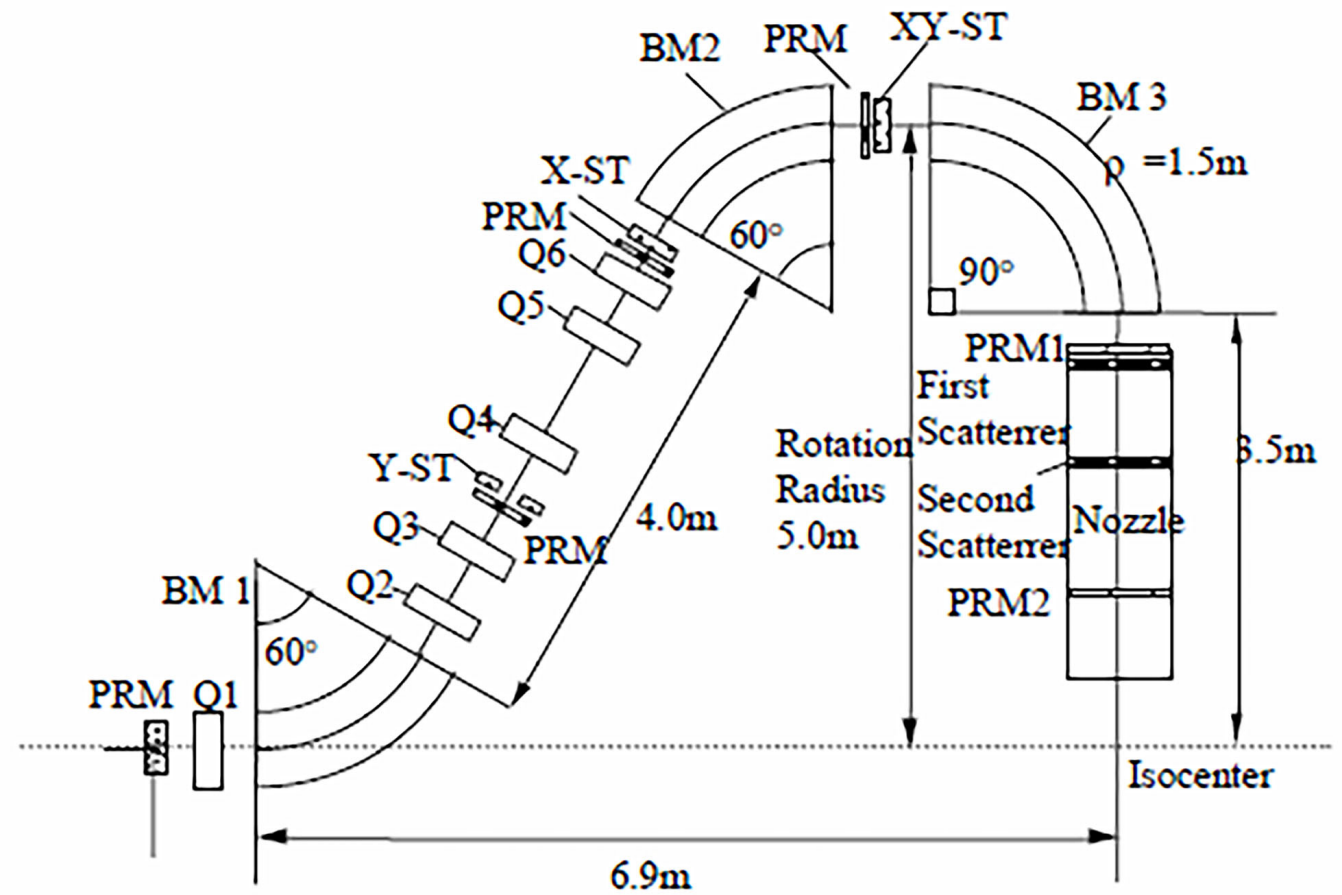
Pavlovic (30) layout of magnets in an ion therapy gantry – after (5). In this example two bending dipoles (BM 1 and BM 2) offset the beam 5 m from the isocenter and dipole BM 3 bends the beam back towards the isocenter and the patient platform. In other systems BM 1 and BM 2 would have other but equal bend angles [e.g. 45° (19)], and still others may employ only two bending magnets (e.g. BM 1 with 45°or 60° and BM 3 with 135°or150°) to achieve the same result (31, 32).

**Figure 4:**
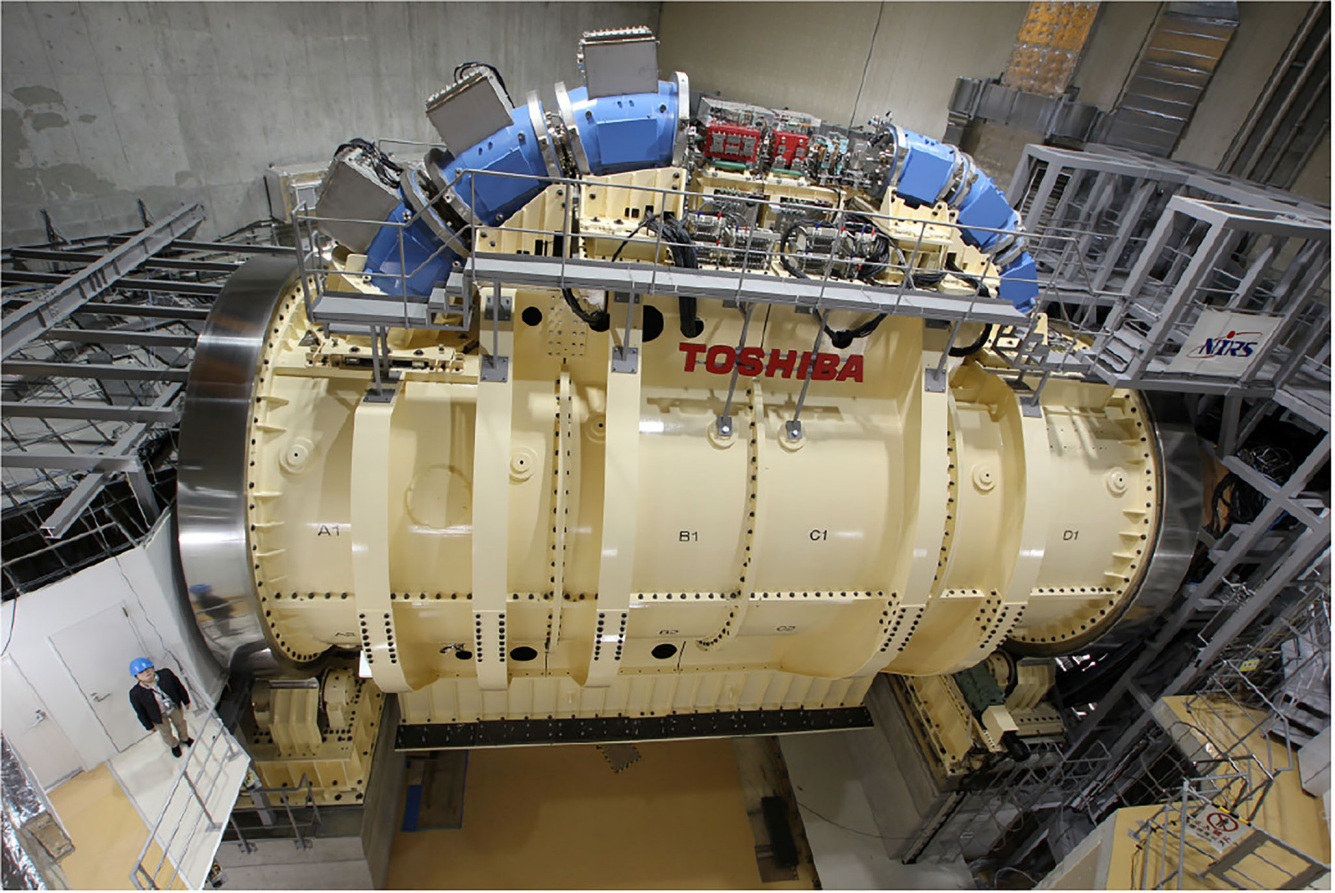
Toshiba’s gantry with superconducting technology capable of 360° rotation about the isocenter – after (33).

In the present context a gantry is a massive structure that rigidly holds in place the guidance magnets of ion beam therapy. A typical magnet arrangement is shown in **Figure 3**. The figure also indicates the so-called “isocenter” or axis about which the gantry rotates thereby enabling the ion beam to enter the tumor in all directions. The term “gantry” may refer to the mechanical structure, the magnet string (5), or the entire system as illustrated in **Figure 4**.

### 5.3 Components of the Magnet String and Gantry

**Figure 3** illustrates a Pavlovic-type (30) arrangement of magnets and devices in an ion therapy gantry. The beam is bent by three dipole magnets BM 1, BM 2, and BM 3, and kept in focus by six quadrupole magnets Q1 to Q6. The beam enters the gantry through a monitor PRM that monitors and records the center of the beam and its size. Three other PRMs are associated with steering magnets for beam trajectory correction and two more, PRM1 and PRM2, are located in the nozzle. Several nozzle arrangements have been used to passively or actively spread the mm-size beam over a treatment area that may be as large as 30 cm^2^ (5, 34). Described in detail by (31) are: (i) uniform scanning nozzles, (ii) pencil scanning nozzles, (iii) single scattering nozzles, and (iv) double scattering nozzles. The latter version is represented in **Figure 3**.

### 5.4 Bending Dipoles: Momentum Acceptance

During a typical treatment session a proton beam energy may need to be varied from 70 to 250 MeV. This produces a change in rigidity of from 1.231 to 2.431 Tm requiring the field of a 1-m-radius dipole to track the beam energy and hence increase from 1.23 to 2.43 T. In the absence of field-change a typical normal dipole will accept a beam momentum change, *dp/p*, of less than 1% ( (35), p.2). Tumors are typically scanned in layers 5 mm deep enabled by a momentum sweep of 1% ( (35), p.4), the layer-scan taking of order 100 ms. A *dp/p* of 1% corresponds to a beam energy change *dE/E* = 2*dp/p* = 2%. Thus to control a 100 MeV beam (proton magnetic rigidity 1.483 Tm) a 1-m-radius dipole would require a field change of 15 mT. On this basis the average sweep rate of the scan is 150 mT/s (20 times faster than LHC). Taking another approach, a *dE/E* of 2% implies 50 scanning steps covering the energy range from 70 to 250 MeV and a 1-m-radius dipole field range of 1.23 to 2.43 T. At 100 ms/step this leads to an average sweep rate of 240 mT/s. From another standpoint, at 5 mm per layer a proton-beam-depth range of 10-35 cm would require 50 scanning steps, leading to the same result.

Sweep rates of hundreds of mT/s cause stability and energy-loss problems for superconducting magnets. So when considering a gantry upgrade from normal-conducting to superconducting magnets these high ramp rates created a demand for achromatic bending dipoles with wide momentum acceptances, such *dp/p* = 5-10%, 25%, and 40-50% [(35), p.3]. The use of such magnets, which enable the entire energy range to be covered in just a few steps, eases the ramp-rate requirement.

### 5.5 Normal-Conducting Gantry Magnets

As outlined in (20) the use of normal-conducting iron-core dipole magnets with their maximum bore field, *B*, of about 1.8 T governs the size of most commercial gantries. At a typical 250 MeV proton beam rigidity, *R =* 2.43 Tm the 1.8 T field calls for a bending radius *ρ* = *R/B* = 1.35 m. The addition of 3.5 m for distance from the nozzle entrance to the isocenter leads to a gantry rotation radius of about 5 m (**Figure 3**). Thus proton gantries are about 10-12 m in diameter, 7-10 m long (**Figure 3** also (18)) and weight about 100-200 tons (20). Further details are provided in **Appendix B**.

Much larger than the above proton gantries is the famous carbon-ion gantry of the Heidelberg Ion Beam Therapy Centre (HIT). The maximum treatment beam energy of 425 MeV/u yields a beam rigidity of 6.58 Tm (**Table 6**) which calls for a 1.8 T dipole radius of *ρ = R/B* = 3.65 m. The supporting gantry is ≈ 12 m in diameter, ≈ 21 m long, and the overall system (including 135 tonnes of magnets) weighs ≈ 650 tonnes (36). These statistics signaled the beginning and end of normal-conducting carbon-ion gantries and ushered in the need for superconducting magnets (36). In conclusion we note that HIT should not be confused with HITRI+ which stands for “Heavy Ion Therapy Research Initiative” a design study to assess the relative merits of CT and CCT magnets (Sections 5.7.2 and 5.7.4) for synchrotrons (Section 4.2) and CT/CCT and toroids (Section 5.7.6) for gantries.

### 5.6 Superconducting Gantry Magnets for Proton Therapy

#### 5.6.1 Conventionally Wound Magnets

It is easy to argue that proton gantries are adequately served by normal-conducting magnets. Nevertheless superconductivity has enabled several improvements to gantry and magnet design. Alonso and Antaya (18) considered the size advantage accompanying the substitution of 2 T normal-conducting dipoles with superconducting ones with 4 T or higher fields. The smaller bend radius and smaller size were estimated to reduce the gantry diameter by about 3 m. Alonso et al. have also described a novel gantry concept being developed at the time by ProNova Solutions based on achromatic (9% momentum acceptance) combined-function magnets. In that arrangement a 60° bend was followed by a set of magnets (possibly a pair) contributing to a final 150° bend. The total mass of the magnets was estimated to be less than 5 tons. The “direct replacing” of normal-conducting magnets with superconducting ones should not only lower the weight but also the cost of the gantry. With these advantages in mind Bontoiu and Sanchez-Segovia (37) went on to model a lattice of 36 combined-function superconducting magnets. Inspired by LHC technology the combined-function magnet consists of one layer of quadrupole coils on top of one layer of cos-*θ* dipole coils. Combined-function magnets possess much larger momentum acceptances than do individual dipoles and quadrupoles. In this case a fixed-field beam energy variation of 50 MeV could be accommodated, enabling an energy range of 100 to 250 MeV to be covered in only three steps. The superconducting gantry design study of Wan et al. (38) was motivated not only by size, weight, and cost considerations but also the need for large energy acceptance. Their design achieved an energy acceptance of ± 21% enabling beams of 150- 30 MeV to be bent without field ramping. Wan et al’s compact fixed-field-alternating-gradient (FFAG) gantry, or magnet string, consisted of three groups of seven achromatic 3.2 T superconducting magnets, each group creating a 90° bend (38).

#### 5.6.2 Canted Cosine Theta Magnets

It was shown in 1970 that an overlaid pair of solenoidal coils tilted in opposite directions could generate a dipolar field normal to the solenoidal axis (39). Exploited in recent years by the superconducting magnet group at the Lawrence Berkeley National Laboratory (LBNL) this tilted-double-helix magnet is now referred to as canted cosine theta, CCT. By suitably modifying the winding scheme quadrupoles and higher order multipoles can also be generated (18). Not only that, but a combined-function magnet can be produced by winding a CCT dipole on top of a CCT quadrupole (40). In pursuing CCT technology the LBNL group went on to introduce a new magnet concept – the alternating-gradient canted cosine theta (AGCCT) magnet (35, 40). In this magnet the inner quadrupole winding consists of multiple sections with the current reversed between sections, e.g. 5 sections for a winding designated FDFDF. LBNL’s design can be characterized as fixed-field alternating-gradient since its large momentum acceptance (~ 25%) enables a large energy range to be transmitted without changing the field. The advantages claimed for gantries incorporating the new magnet design are: (i) an order of magnitude reduction in weight, (ii) possible size reduction, e.g. diameter 5 m and length 8.3 m, and (iii) rapid scanning (35). Gantry layouts described by the LBNL group are: (i) three groups of seven achromatic 3.2 T superconducting magnets (with sextupole and octupole components in the middle five of each group), with each group creating a 90° bend (38), (ii) two 75° AGCCT bending magnet groups to offset the beam 2.5 m “above” the exocenter followed by one 90° AGCCT group (35), (iii) one normal-conducting 45 dipole to deflect the beam “upwards” followed by a single 135° bend to guide the beam back to the exocenter (40). As with other superconducting gantry systems the magnets are intended to be conduction cooled.

#### 5.6.3 Superconductors for Magnet Windings

Several practice CCT-based coils were wound at LBNL using parallel stacks (6x1 and 8x1) of insulated square (1.6 mm x 1.6 mm) NbTi wire. During the above studies the LBNL group conducted a detailed evaluation of the advantages and disadvantages of Nb_3_Sn, MgB_2_, and HTS wires and selected NbTi for a number of reasons [(35) **Table 8**, see also (41)]. It is interesting to note that the Toshiba Corporation has been considering HTS magnets with the aim of reducing gantry size and has designed and fabricated a model magnet (42).

**Table 8:** MEVION S250 compact proton therapy systems.

Facility	Location	Status
MedStar Georgetown University Hospital	Washington, DC	Under installation
Stevenson Cancer Center	Oklahoma City, OK	Clinically accepted
UF Health Cancer Center at Orlando Health	Orlando, FL	Clinically operational
Ackerman Cancer Center	Jacksonville, FL (a)	Clinically operational
Seidman Cancer Center, University Hospitals	Cleveland, OH	Clinically operational
Siteman Cancer Center, Barnes Jewish Hospital, Washington University	St Louis, Missouri	Clinically operational
Robert Wood Johnson University Hospital	New Brunswick, NJ	Clinically operational
ZON-PTC at Brightlands Maastricht Health	Maastricht, Netherlands (b)	

(a) S250 operational; S250i under contract, (b) S250i.

#### 5.6.4 The MEVION S250

**Figure 5:**
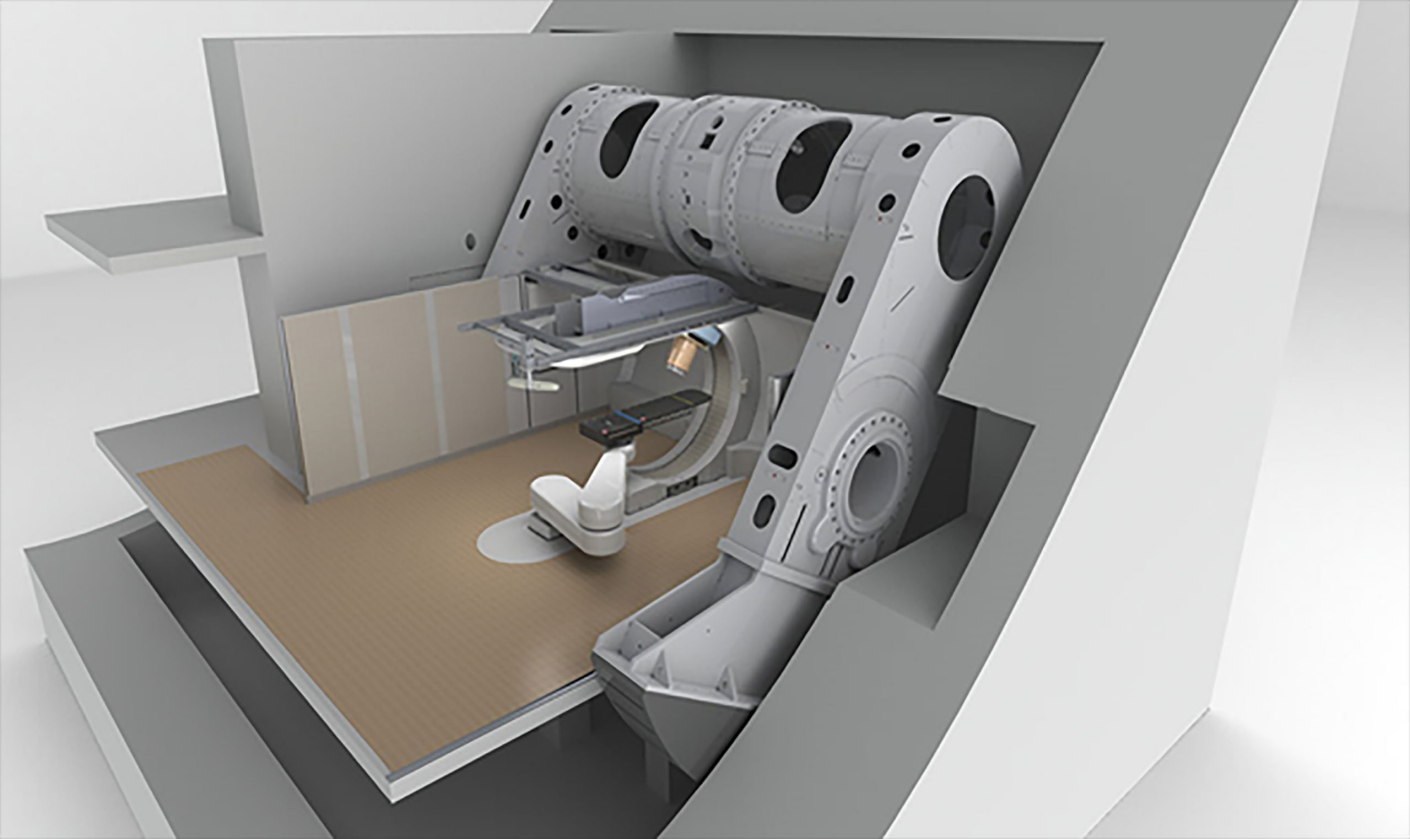
The MEVION S250 gantry-mounted proton synchrocyclotron – after Jongen (7).

The MEVION S250 system is unconventional in that the gantry carries no beam-transport magnets. Instead the proton beam emanates directly from a gantry-mounted 250 MeV superconducting synchrocyclotron whose magnet, wound with Nb_3_Sn wire, and cryocooled to 4 K, delivers a central field of 9 T. Weighing only 22 tons Mevion’s SCS is much lighter than comparable machines, e.g. IBA’s 50 ton 230 MeV superconducting synchrocyclotron (see **Figure 5**). At least 7 S250 systems are presently operational and the S250i with pencil beam scanning is also available, **Table 8**.

### 5.7 Superconducting Gantry Magnets for Hadron Therapy

#### 5.7.1 Background

Freed from the need for an iron core, superconducting magnets can be much stronger in field and lighter than their normal-conducting counterparts and hence are suitable as hadron-therapy gantry magnets. Whereas a 230 MeV proton beam can achieve a dose depth of 33 cm, carbon ions need to be accelerated to 400 MeV/u (4.8 GeV). Accordingly the magnetic rigidities (*M = ρB*) of these beams are 2.32 and 6.35 Tm, respectively. The dipole field needed to achieve a 1.5 m 230 MeV proton-beam bend radius is 1.5 T whereas the 400 MeV/u carbon-ion beam requires 4.2 T (**Table 6**), achievable only in a superconducting magnet. Such magnets can provide very small carbon-beam bend radii; given that *ρ* = 6.35/*B* radii of less than 1 m are possible.

The advantage of carbon ions is that, being heavier than protons, they provide a higher RBE. After their pioneering work with proton beams in 1954 LBNL moved on to helium ions in 1957, and neon ions in 1975, but ended all radiotherapy programs in 1992 (15). In 1994 carbon ion radiotherapy (CIRT) was picked up by Japan’s National Institute of Radiological Sciences (NIRS) using the Heavy Ion Medical Accelerator in Chiba (HIMAC). Other centers were soon to follow; five in Japan and others in Austria, China, Germany and Italy ( (7, 15) and **Table 4**). In spite of its innovative pioneering work the US does not house a single CIRT center, although initial planning has been announced for the establishment of such centers in California and Colorado [(43, 44) see also (45)].

#### 5.7.2 Conventionally Wound LTS Magnets for Carbon Therapy

In 2012 Iwata et al. and others from the Toshiba Corporation reported on the design of a rotating gantry system with superconducting magnets for carbon ion therapy (46). Then in 2013 Toshiba received an order from NIRS for such a system, the world’s first (33). The cylindrical gantry is depicted in **Figure 4**. In addition to a pair of scanning magnets, three pairs of steering magnets and beam profile monitoring magnets it consisted of 10 superconducting combined-function bending magnets. One of the design goals was to achieve a compact superconducting gantry, much smaller than HIT’s room-temperature one and comparable in size (length 13 m, radius 5.5 m) to existing proton gantries. The 10 bending magnets, BM1-10, of the Toshiba/NIRS 430 MeV/u magnet system were arranged in 3 groups – BM1-3, BM4-6 and BM7-10, **Table 9**.

**Table 9:** Some properties of the toshiba/NIRS superconducting dipoles (46).

Magnet Group	BM1-3	BM4-6	BM7-10
Bend angle, degrees	70	70	90
Bend Radius, *ρ*, m	2.3	2.3	2.8
Max Dipole Bore Field, *B*, T	2.88*	2.88	2.37

*Magnetic rigidity of 430 MeV/u carbon ions, M = 6.627 Tm (**Appendix A2**) hence B = M/ρ =2.88 T.

The superconducting combined-function coils have a layered structure: the innermost 8 layers being cos(*2θ*) quadrupoles and the outer 26 layers being cos*θ* dipoles. The small momentum acceptance of the system necessitated the use of low AC-loss NbTi wire – 10 μm filament diameter with CuNi barriers (47). The magnets were conduction cooled with 1.5W/4.2K Gifford-McMahon cryocoolers. Three sets were mounted on each of BM1-6 and four sets on each of BM7-10 for a total of 34 cryocoolers (47). An even more compact gantry was described by Iwata et al. (48). Consisting of only three identical 90° combined-function magnets it was only 5.1 m in length and 2.5 m in radius. Designed for 430 MeV/u carbon ions and hence a beam rigidity of 6.627 Tm each magnet has a bending radius *ρ* = 1.32 m and a maximum bore field *B* = 5.02 T. As described and depicted in (48) the combined-function magnet consisted of a 20-layer of cos*θ* dipole winding on top of a 6-layer cos(*2θ*) quadrupole winding.

#### 5.7.3 Conventionally Wound HTS Magnets for Carbon Therapy

In 2013 Toshiba began the development HTS magnets one of its aims being to further reduce the size of heavy-ion-therapy gantries (47). Based on Toshiba’s above low-temperature-superconducting gantry an HTS gantry was designed and model magnet designed, built, and tested. Selected for the windings was a REBCO HTS tape 4 mm wide and 0.1 mm thick. Wound with 820 m of tape, the magnet consisted of 24 saddle-shaped REBCO coils stacked in four layers and attached to an iron yoke (47). Conduction cooled to about 4 K by a GM cryocooler the magnet generated a bore field of 1.2 T.

#### 5.7.4 Canted Cosine Theta Magnets for Carbon Therapy

As mentioned in Section 5.5 at the Heidelburg Ion-Beam Therapy Center (HIT) the 135 tonnes of normal-conducting magnets are supported by a 21 m long, 6 m radius, gantry weighing an additional 515 tonnes. The resulting demand for a smaller lighter gantry called for introduction of superconducting magnets. Numerous such systems furnished with conventionally wound cos*θ* and cos(*2θ*) dipoles and quadrupoles have been designed and/or constructed, e.g. (33, 46–48).. To still further reduce size and weight the CCT winding was introduced. Numerous reports describe the implementation of CCT winding within the context of proton therapy [e.g. (35, 40)] but the concept should be applicable to both proton and carbon systems.

Robin et al. described a superconducting magnet string for a compact carbon-ion therapy gantry (49). Their gantry 9.97 m long and 3.12 m radius carries two 45° bending dipoles, numerous quadrupoles, and a 90° large aperture final bending magnet. In a paper that focused attention on the 5 T 90° final bending magnet the authors pointed out that the CCT concept could be applied to dipoles, quadrupoles, and bent magnets (“toroids”). Winding schemes to produce the correct combination of dipolar and multipolar fields were developed. The magnet layout of Kim et al. (19) is identical to that of Robin et al. (49). This work also focused attention on the large aperture 90° final bending magnet recognizing that the field to bend 430 MeV/u carbon-ion beam (*R* = 6.627 Tm) to a radius of 1.269 m is 5.22 T. Sextupole components in the fringe field region of the dipole were removed by adjusting the coil winding in the main body of the dipole.

#### 5.7.5 Recent Initiatives in Magnets and Gantries

IFAST (Innovative Fostering of Accelerator Science and Technology, a CERN project) was the subject of a meeting convened by L. Rossi (INFL, Milano) that set out to prepare a proposal dealing with Work Package WP-8 (innovative superconducting magnets (L. Rossi)) and WP-4 (magnet design) (50). The objective of WP-4 included engineering design for the HITRI accelerator magnets (see Section 5.5) and the HITRI gantry magnet.

#### 5.7.6 Superconducting Toroidal Gantry for Hadron Therapy

Bottura, Felcini, et al. (CERN) described the magnetic design of a superconducting gantry in the form of a torus configured for proton or hadron therapy. For the windings of this “GaToroid” both LTS (NbTI) and HTS (REBCO) options were considered. The device eliminates the need for rotating structures and operating at constant current and magnetic field is able to deliver a beam at discrete angles over a wide range of energies, e.g. 70 MeV to 250 MeV.

## 6 Therapy Systems

### 6.1 Magnets and Gantries

The designers of ion-beam therapy systems are continually striving for improved beam optics, smaller size, and lower cost. Regarding optics, pencil-beam scanning was early introduced by the vendors. Introduction of the alternating gradient CCT magnet with its large momentum acceptance (~ 25%) enabled large energy ranges to be transmitted at fixed field, thus reducing the need for low-AC-loss magnet windings. Therapy systems consisting of large accelerators, beam lines and gantries are large and expensive. Superconductors went a long way towards reducing their size. Another important step was taken by MEVION which, by mounting a compact 17 ton synchrocyclotron on the gantry frame, eliminated the need for a string of quadrupoles and bending dipoles. Although the MEVION could be fitted with the desired pencil-beam scanning system, the fixed 250 MeV of the synchrocyclotron called for graphite energy degraders to vary the bean energy. A potential improvement would be to introduce the MIT-ProNova-designed 5 ton ironless variable-energy (70-230 MeV) synchrocyclotron.

Sections 5.5 through 5.7 have outlined the evolution of magnet systems and gantries from proton to carbon-ion, from normal-conducting to superconducting, from standard cos*θ* and cos(*2θ*) windings, to tilted double-helix (CCT) windings. We see proton magnet system weights decreasing from an estimated 20~40 tons to less than 5 tons. Gantry layouts have been simplified and magnets have been improved, for example: (i) Bontoiu’s proton gantry (37) consisted of a string of 36 combined-function superconducting magnets, (ii) Toshiba’s carbon-ion gantry consisted of 10 combined-function superconducting magnets arranged in 3 groups for bends of 70° (3 magnets), 70° (3 magnets), and 90° (4 magnets) (18, 33, 46) (47), (iii) Another Toshiba carbon-ion gantry consisted of just 3 superconducting combined function 90° bend magnets (48), (iv) substitution of the standard cos*θ* and cos(*2θ*) windings by tilted double-helix (CCT) windings, for which several advantages have been claimed viz: (a) an order of magnitude reduction in weight, (b) possible gantry size reduction, e.g. diameter 5 m and length 8.3 m, and (c) rapid scanning (35).

### 6.2 Magnet Windings

#### 6.2.1 LTS Windings

Wan et al. (35) (LBNL) have reviewed the suitability of the well-known low temperature (LTS) and high temperature (HTS) superconductors for gantry magnet windings and conclude that for their proton gantry application NbTi was the conductor of choice. For the windings of their CCT combined-function magnets Wan et al. selected “SSC-inner” NbTi strand 0.8 mm in diameter, filament diameter 6 μm. For one of the magnets the 8 conductors in the two dipole layers will be powered in series and the 26 conductors in the two quadruple layers will be separately powered in series. The suitability of NbTi has also been claimed by others, for example: (i) the 10 magnets of a Toshiba gantry (46) are wound with 0.9 mm diameter NbTi wire, (ii) the 3 combined-function magnets of another Toshiba gantry (48) are wound with 0.92 mm NbTi wire twisted to 6 + 1 for a final cable diameter including insulation of 3.0 mm; the coil currents were 710 A (quadrupole) and 920 A (dipole), (iii) another LBNL magnet (40) was wound with series-connected parallel stacks (6x1 and 8x1) of 1.6 mm square insulated insulated NbTi wires.

#### 6.2.2 LTS and HTS Windings

With a magnetic rigidity of 6.627 Tm a 430 MeV carbon-ion beam can be bent to a radius of 1 m by a dipole with a bore field of 6.6 T (although the field-at-winding will be higher than this to an extent that depends on magnet design). Wan et al’s (35) review of some of the key properties of the wire-formable LTS and HTS superconductors reminds us that with 4.2 K upper critical fields (*B_c2_*) of 10.5-11 T (NbTi), 19-27 T (Nb_3_Sn), 9-10 T (MgB_2)_, 70-75 T (Bi_2_Sr_2_CaCu_2_O_8+x,_ i.e. “Bi:2212”), and 92-95 T (YBa_2_Cu_3_O_7-x_, i.e. “REBCO”) they all apparently qualify from a 4.2 K critical field standpoint. Down-selection then involves consideration of other materials properties, magnet fabrication problems, and cryogenic issues. After winding *(W*) with Nb_3_Sn wire the magnet needs to be reacted (*R*) for ~160 hours at temperatures up to 650°C and Bi:2212 coils require ~125 hours at 888°C. MgB_2_ requires a relatively mild heat treatment (60 min/675°C) and either *W&R* or *R&W* coils can be produced. From a manufacturing standpoint MgB_2_ is more attractive than Nb_3_Sn and Bi:2212 but its critical field does not leave enough margin to enable conduction cooled operation at the above field strengths. NbTi and REBCO wires in the as-received condition are suitable for coil winding; this is a great advantage. The selection in favor of REBCO, dictated by cryogenic issues, is discussed in the following sections.

### 6.3 Cryogenics

#### 6.3.1 Gantry Cooling

The accelerator magnets of high energy physics are wound with high-current-carrying Rutherford cable to maintain a high ampere-turn ratio while minimizing the magnet inductance. Different considerations govern the choice of magnet design and conductor size in superconducting particle beam gantries. Inductance is not an issue but magnet current has to be relatively small to minimize current-lead heat leak into the cryostat. Since the magnet heat load scales with ramp rate a large momentum acceptance (especially if it allows fixed-field operation) is beneficial for thermal design (40). Because the gantry needs to rotate, magnet cooling by liquid helium is not feasible. Instead all superconducting gantry systems are cryogen-free, conduction cooled by way of high conductivity links to cryocooler cold heads. Numerous cold heads may be connected in parallel to various parts of the magnet system (35). The final 90° dipole of a joint IBA/CEA Saclay study was cooled by 10 Sumitomo cryocoolers (18). Mounted on the Toshiba heavy-ion gantry referred to above (47) are 3 groups of 10 bending magnets, BM1-3, BM4-6 and BM7-10. They are conduction cooled with 1.5W/4.2K Gifford-McMahon cryocoolers – three sets on each of BM1-6 and four sets on each of BM7-10 for a total of 34 cryocoolers (47). At a Sumitomo (SHI) model RDK-4150 (1.5W/4.2 K) cold head weight of 18.5 kg (40.8 lb) 34 cryocoolers would contribute about ¾ ton to the weight of the gantry. A lighter gantry could be enabled by circulating gaseous or supercritical helium through the magnets from an off-gantry-mounted cooling and pumping system (51–54) or by implementing a cryogenic oscillating heat pipe system (55).

#### 6.3.2 The Choice of REBCO Conductor for Cryogen-Free Carbon-Ion Gantries

In 2016 Iwata et al. and others from the Toshiba Corporation reported on the design of a compact gantry for 430 MeV/u carbon ions (48). Each of the three 90° dipoles was to have a bend radius of 1.32 m and hence a dipole field strength of 5.02 T. The results of the design study did not include cryogenics and provided no indication of the feasibility of maintaining a field of 5 T under cryogen-free conditions. On the other hand the Toshiba group did report on the design (46, 47), test results (47), and delivery to NIRS of a heavy ion rotating gantry. As reported above (section 5.7.2, **Table 9**) the three sets of NbTi-wound bending magnets had maximum fields of 2.88 T, 2.88 T, and 2.37 T. It is doubtful if fields much higher than this can be achieved in commercial conduction-cooled systems. Attempts to build NbTi-wound conduction-cooled magnets for magnetic resonance imaging (MRI) have not succeeded, while recent research and development of conduction-cooled 3 T MRI systems has focused on MgB_2_. But for ion-beam therapy, to avoid the difficulties associated with reaction-heat-treatment and to gain the advantages of its high *T_c_*, *B_c2_*, and engineering critical current density, *J_E_*, and cryogenic stability, REBCO is arguably the conductor of choice.

The 4.2 K, 5 T the *J_E_* of “standard” SuperPower REBCO tape at 4.5 x 10^3^ A/mm^2^ (B//) (56) is 4 times greater than that of NbTi. An even higher *J_E_* (5.4 x 10^4^ A/mm^2^) is becoming obtainable as a result of: (i) increasing the Zr doping content from 7.5% to 25% (x3), (ii) increasing the film thickness (x2), (iii) reducing the tape thickness from 100 μm to 50 μm (x2). As a result the 4.2 K, 5 T *J_E_* of REBCO tape is more than 40 times that of NbTi. One reason for the rejection of REBCO was that tape geometry was not regarded as suitable for magnet winding. This is no longer a problem. REBCO is now available in wire form - thin tapes spirally wrapped along a wire core 0.51-0.8 mm in diameter.

## 7 Prospects for Improvement

### 7.1 Need for Image Guidance

To perform an efficient treatment accurate image guidance techniques that can distinguish a tumor from healthy tissue are required. Imaging is associated with target accuracy, of targeting, dosimetry, treatment outcome assessment, and prognosis. Images are used in radiation therapy during the following processes: (1) offline treatment planning – tumors and other anatomical structures are identified and the treatment is simulated on a treatment planning computer. During this process CT, MRI and even PET-CT or other type of images are commonly used; (2) imaging is applied to set up the patient in the treatment room and to convert the virtual treatment (a treatment plan) into the real treatment; (3) images are used to assess the treatment outcome: effectiveness, toxicities, and prognosis, etc. While Processes 1 and 3 are usually carried out offline, Process 2 is often performed with the imaging device attached to the treatment machine inside the treatment room to align the patient in the treatment position or during the treatment to monitor the targeting of tumors and organs-at-risk. Process 2 is complex and needs to conform to the gantry and the beam delivery system. The currently available image-guided particle therapy systems are mainly based on 2D orthogonal X-ray imaging, in-room 3D computed tomography (CT) or on-board cone-beam CT (CBCT) imaging. However, 3D or 4D CT and MRI images are commonly used off-line for treatment planning (57). Imaging technologies such as the proton Computed Tomography (or pCT) (58, 59) or MR-guided proton therapy have been proposed but are not yet available for clinical use (60). While X-ray based imaging has difficulty resolving soft-tissue the converse is true for MRI-guided particle therapy. MRI guidance in photon (radiation) therapy MRgRT, which entered clinical practice not long ago, gradually became popular during the past decade (61). Currently there are several commercial available MRgRT manufacturers in the market: ViewRay (62), Elekta (63), and Varian Medical Systems/IMRIS (64).

**Figure 6:**
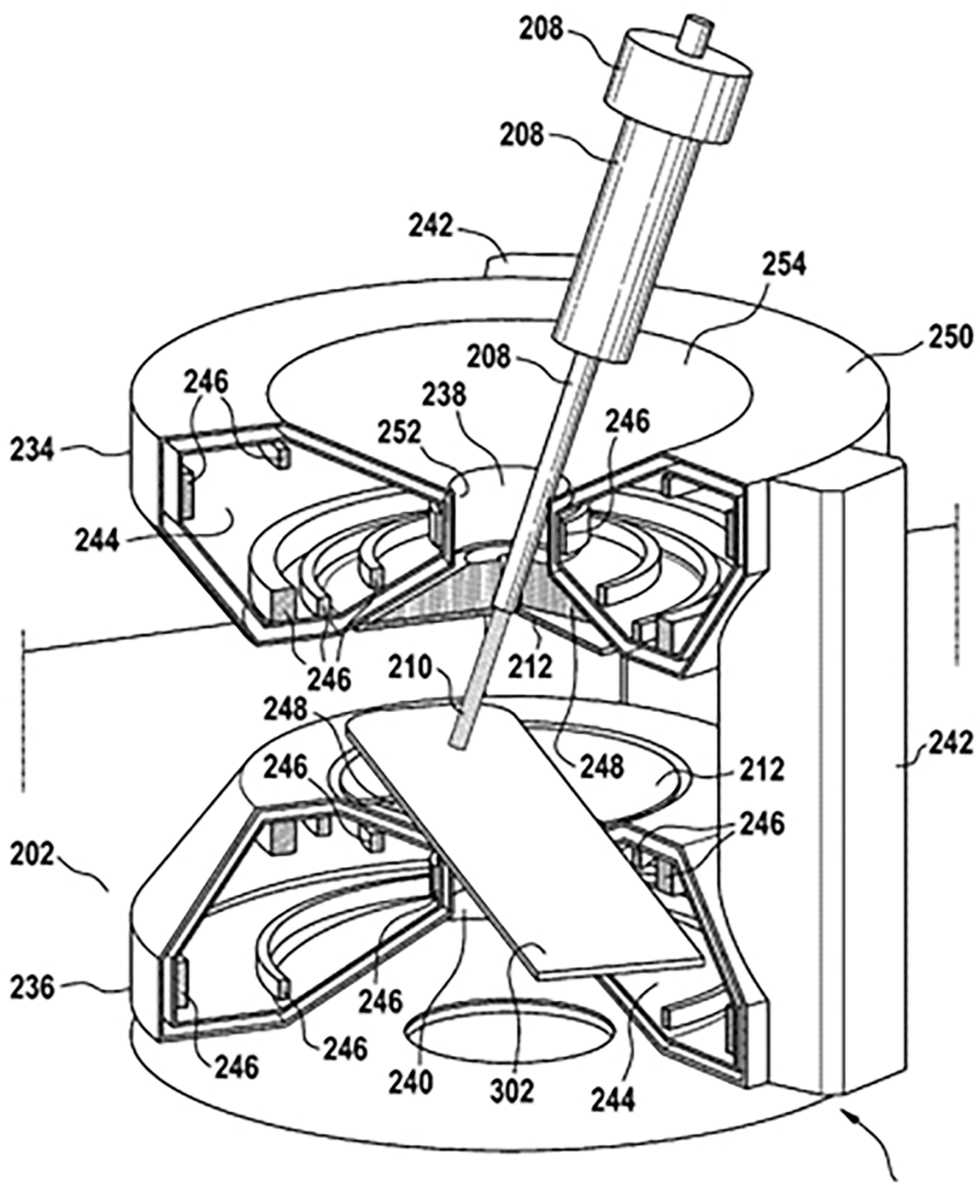
Possible configuration of a hybrid MRI proton system, taken from patent application US 10 , 173 , 077 B2 (45). Date of Patent: Jan. 8, 2019 (67).

In order to take advantage of the peak effect (discussed in Section 2.1) the exact depth of the tumor (the target) must figure into the treatment. An error in the target depth of a few mm that may result in only a few percent change in the photon dose may lead to a 100% change in the proton dose (67). This emphasizes the need for direct visualization of the tumor position. Such “image guidance” using MRI has been proposed and modeled by numerous researchers (see section 7.3). The principle of MRI guided proton therapy is illustrated in **Figure 6**.

### 7.2 MRI and Proton Therapy

**Figure 7:**
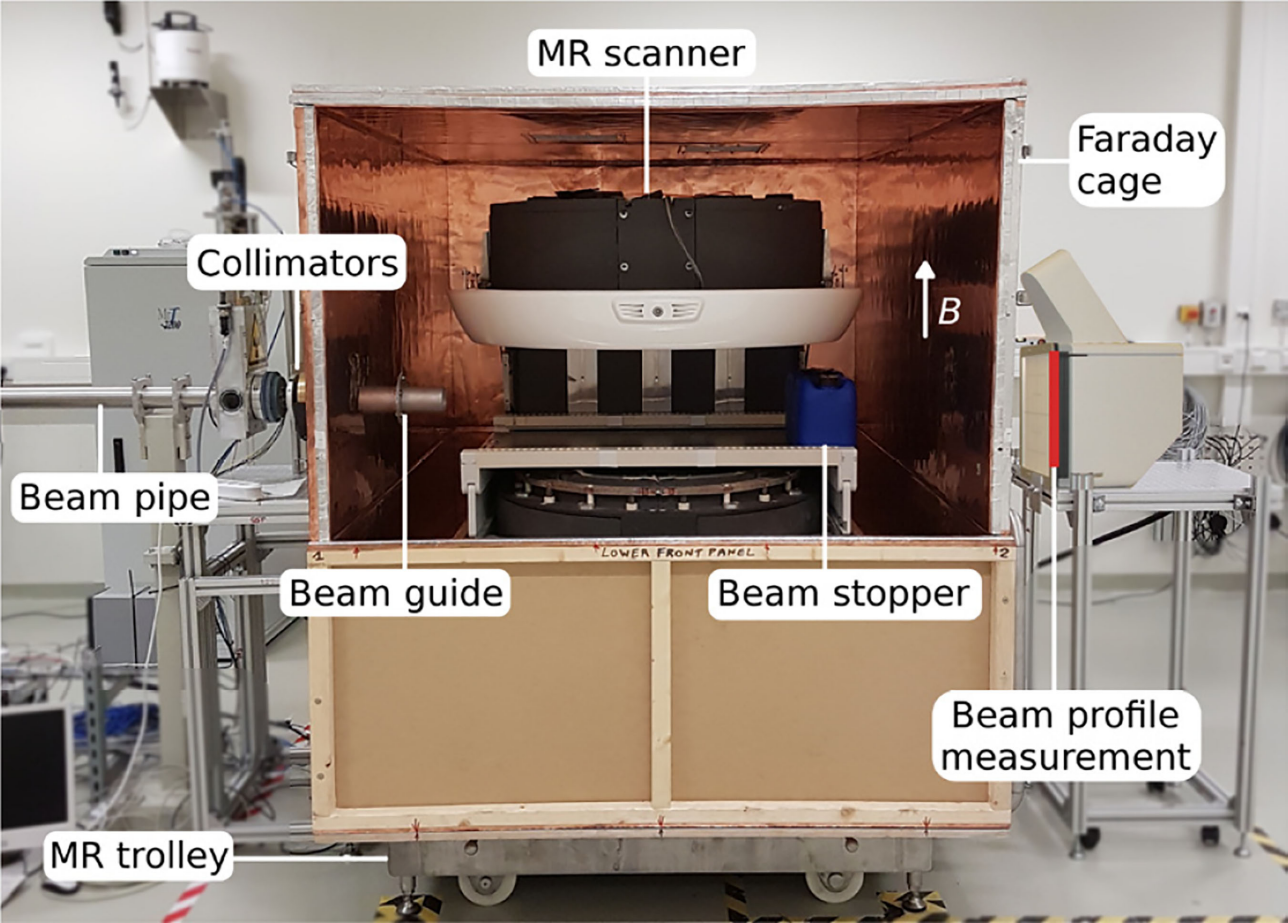
The prototype MR-integrated proton therapy system at the fixed horizontal beam line in the experimental room of the Dresden proton therapy system – *after* (65, 66).

Photon irradiation requires a well-defined beam aimed in the right direction. In proton therapy it is also necessary to predict ensure that the beam will terminate at the tumor side. The additional margin needed for range uncertainty detracts from the benefit of proton therapy. Image guidance is introduced to overcome this uncertainty (67). In a combined MRI-proton system it is necessary to consider mutual electromagnetic interaction between the proton beam and the MRI field. For example Monte Carlo computer simulations and measurements have revealed that a 190 MeV proton beam would be deflected by about 1 cm upon entering a 1 T field. Such effects would influence treatment planning and dose delivery. External magnetic fields that could influence MR image quality come from sources such as: (i) the proton generating cyclotron, (ii) the gantry beam line and steering magnets; the fringe field of such magnets, which can be up to 100 µT, could detrimentally affect the MR image quality. To further investigate such effects Hoffmann and his team (65, 66), in association with OncoRay, attached an open 0.22 T MRI scanner to a fixed horizontally mounted proton research beam line, **Figure 7**.

In a useful experimental study Inaniwa et al. (68) investigated the effects of magnetic fields (0.3 and 0.6 T) applied transversely (*B_T_*) and longitudinally (*B_L_*) a proton beam adjusted to deposit energies of 1.1 and 3.3 keV/µm into normal and cancer cells. Effectiveness was gauged by the index *R_10_ ≡ D_WO_/D_W_* which is the ratio of the dose that would result in a survival fraction of 10% in the absence (*D_WO_*) and presence (*D_W_*) of the magnetic field.

For cancer cells exposed to 1.1 keV (3.3 keV) proton beams *R_10_* increased to 1.10 (1.11) and 1.11 (1.12) in longitudinal magnetic fields (*B_L_*) of 0.3 T (0.6 T).

For normal cells *R_10_* increased to 1.13 (1.17) and 1.17 (1.30) in these longitudinal magnetic fields.

For both normal and cancer cells *R_10_* showed no significant change in the transversely applied fields

Inaniwa et al. pointed out that the longitudinal field enhancement effect should be taken into account in the design of an MRI-proton system (68).

### 7.3 Experiment and Modeling

No commercial scale versions of the research system described in **Figure 7** can be made available for experimentation. However numerous modeling studies have been undertaken on the general topic of beam-line/magnetic-field interactions (see **Table 10**, Bibliography). Oborn et al. (69) have modeled the path of a proton beam through the 3D field of a 1 T split bore MRI magnet. Used for the modeling was the software GEANT4 (GEometry ANd Tracking), a platform for simulating the passage of charged particles through matter using a Monte Carlo method. Significant rotation of the beam was observed in the longitudinal orientation while a more complex path was seen in the transverse field. It was concluded that pencil beam scanning was favored for either longitudinal or transverse field orientations.

**Table 10:** Bibliography on MRI-Guided Ion-Beam Therapy.

B.M. Oborn, S. Dowdel, P.E. Metcalf, et al., “Future of Medical Physics: Real-time MRI-guided proton therapy” *Med. Phys.* 44 77-90 (2017)
L.N. Burigo nd B.M. Oborn, “MRI-guided proton therapy planning: accounting for an inline MRI fringe field”, *Phys Met Biol* **64** 215015 (2019)
A. Hoffmann, B. Oborn, M. Moteabbed, et al, “MRI-guided proton therapy: a review and a preview”, *Radiat Oncol* **15, **129 (2020)
T. Freeman, “MRI-guided proton therapy: a status update”, *physics world* (2019)
S.E.M. Huijsse, A. Knopf, L.V. van Dijk, et al “MR-only guided proton therapy: advances, future perspectives and challenges”, *MReadings:MR in RT*, pp. 16-21
B.M. Oborn, S. Dowdell, P.E. Metcalf, et al, “MRI Guided Proton Therapy: Pencil beam scanning in an MRI fringe field”, *Radiother Oncol.* **118**(Supplement 1):S78–9 (2016)
B.M. Oborn, S. Dowdell, P.E. Metcalf, et al,”Proton beam deflection in MRI fields: Implications for MRI-guided proton therapy: *Medical Physics* **42**, 2113 (2015)
B.M. Oborn, S. Dowdell, P.E. Metcalf, et al,”Future of Medical Physics: Real-time MRI guided Proton Therapy”, *Med Phys.* **44** 77–90 (2017)
M. Moteabbed, J. Schuemann, and H. Paganetti”, Dosimetric feasibility of real-time MRI-guided proton therapy” *Medical Physics*, **41** 111713 (11pp) (2014)
J Hartman, C Kontaxis, G H Bol, et al. “Dosimetric feasibility of intensity modulated proton therapy in a transverse magnetic field of 1.5 t.” *Physics in Medicine and Biology*, **60** 5955 (2015)
J. Hartman, J.J.W. Lagendijk, B.W. Raaymakers, et al., “MRI guided proton therapy”, *UMC Utrecht*
B.W. Raaymakers, A.J. Raaijmakers, and J.J. Lagendijk, “Feasibility of MRI guided proton therapy: magnetic field dose effects”, *Phys Med Biol.* **53** 5615–22 (2008)
C. Kurz, G. Landry, A.F. Resch et al., “A Monte-Carlo study to assess the effect of 1.5 T magnetic fields on the overall robustness of pencil-beam scanning proton radiotherapy plans for prostate cancer”, *Phys Med Biol.* **62** 8470–8 (2017)
H. Fuchs, P. Moser, M. Gröschl, et al., “Magnetic field effects on particle beams and their implications for dose calculation in MR-guided particle therapy”, *Med Phys*. **44** 1149–56 (2017)
S.M. Schellhammer and A.L. Hoffmann, “Prediction and compensation of magnetic beam deflection in MR-integrated proton therapy: a method optimized regarding accuracy, versatility and speed”, *Phys Med Biol.* **62** 1548–64 (2017)
S.M. Schellhammer, A.L. Hoffmann, S. Gantz, et al. “Integrating a low-field open MR scanner with a static proton research beam line: proof of concept”, *Phys Med Biol* **63** 23LT01 (2018)
C.M. Rank, N. Hünemohr, A.M. Nagel, et al., “MRI-based simulation of treatment plans for ion therapy in the brain region”, *Radiother Oncol.* **109** 414–8 (2013)
R. Wolf and T. Bortfeld, “An analytical solution to proton bragg peak deflection in a magnetic field”, *Med Phys* **57** N329-N337 (2012)
S.M. Schellhammer, A.L. Hoffmann, S. Gantz, et al., Integrating a low-field open MR scanner with a static proton research beam line: proof of concept”, *Phys Med Biol.* **63** LT01 (2018)
G.G. Marmitt, M. van Goethem, M. Meijers, et al., “Experimental validation of magnetic field deflections of proton beams for online MR-guided PT”, *6th MR in RT Symposium*, Utrecht, July 1^st^–3^rd^ (2018)
R. Wolf and T. Bortfeld, “An analytical solution to proton bragg peak deflection in a magnetic field,” *Phys. Med. Biol*. 57(17), N329–N337
T. Inaniwa, M. Suzuki, S. Sato, et al. “Effect of external magnetic fields on biological effectiveness of protonbeams”, *Int. J. Radiation Oncology. Biology. Physics :BIOLOGY CONTRIBUTION*, **106** 597-603 (2020)

### 7.4 Recommendations

MRI guidance should be introduced in order take advantage of proton over photon therapy. In so doing the influence of external magnetic fields emanating from the imaging and beam guidance systems on image quality needs to be investigated. In so doing it would be useful to produce an engineering design for a full-scale proton-beam/MRI system (e.g. **Figure 6**) using GEANT-Monte-Carlo method modeling. In addition (or alternatively) a small-scale experimental prototype MRI-integrated proton therapy system (e.g. **Figure 7**) could be constructed and used for investigating beam-line/magnetic-field interactions. Given that longitudinal field dose enhancement and other such effects exist, experiments using such a system could be designed to investigate the underlying mechanisms.

To build an effective image-guided or MR-guided proton or particle therapy system, a comprehensive and sophisticated design of accelerator, beam-line, gantry, magnets, and the imaging components needs to be carefully calculated so that all sub-systems could be integrated into a unit that operates efficiently and meets the clinical requirements while reducing cost to a minimum.

## 8 Summary

The paper begins by emphasizing the clinical and commercial importance of proton and carbon ion (in general, hadron) therapy and refers to the manufacturers of such systems of which more than 120 are installed or are under construction worldwide. A general review of charged particle therapy systems refers to six manufacturers and provides in tabular form some details of systems installed in the US, Europe, Asia, and elsewhere. The principles of particle beam therapy are described in terms of the Bragg peak and the spread-out Bragg peak (SOBP) and a comparison is made of the therapeutic properties of photons (x-rays) versus proton-beams and the latter versus carbon-ions beams. An introduction to particle-beam acceleration is followed by descriptions of normal-conducting and superconducting (SC) cyclotrons (including the isosynchronous cyclotron and the synchrocyclotron) and of synchrotrons. An interesting case study describes the evolution of a normal-conducting 220 ton cyclotron into an iron-free SC synchrocyclotron weighing only 5 tons.

The principles of gantry design and the components of the magnet string are outlined. Particle-beam guidance is described in terms of the beam’s magnetic rigidity and the relationship of bend radius, *ρ*, to bending-dipole field strength, *B*. The development of the needed SOBP requires a range of particle-beam energies, typically proton energies of 70-240 MeV. The associated rapid field-sweep would be difficult for SC dipoles unless dipole design allows for broad momentum acceptance. This statement introduces a detailed discussion of gantry magnets for both proton therapy and carbon-ion therapy.

First to be described is the use of normal-conducting iron-core gantry magnets whose 1.8 T bore field calls for bend radii of 1.35 m (250 MeV protons) and 3.65 m (425 MeV/u carbon). Thus although proton gantries are adequately served by such magnets their use in a one-of-a-kind carbon gantry has led to an unacceptably large structure. Both proton and carbon gantries benefit from the use of SC magnets. The “direct replacing” of normal-conducting magnets with SC ones is expected to lower the weight and cost of the gantry.

Several winding arrangements are described. Conventional windings are the familiar cos*θ* dipolar and cos*2θ* quadrupolar windings of high energy particle physics. In the present context they have given rise to the combined-function winding in which a cos*2θ* quadrupole coil is wound on top of a cos*θ* dipole coil. Such magnets possess much larger momentum acceptances than do individual dipoles and quadrupoles and enable beams of a wide range of energies to be controlled by only a few field steps. The use of conventionally wound SC magnets in both proton and hadron therapy is reviewed. Then an alternative winding, the tilted double helix now referred to as the canted cosine theta (CCT) winding, is introduced A combined-function version of it is produced by winding a cos*θ* dipole on top of a cos2*θ* quadrupole. Next to be described is a new magnet concept – the alternating-gradient CCT (AGCCT) – in which the inner quadrupole consists of multiple sections with the current reversed between sections. This design can be characterized as fixed-field alternating-gradient since its large momentum acceptance enables a large energy range to be transmitted without changing the field. Finally, mention is made of a novel CERN-designed superconducting toroidal gantry for hadron therapy, GaToroid. This device, operating under steady state current and magnetic field, is able to deliver a beam at discrete angles over a range of treatment energies.

Cooling of SC rotating gantry magnets is an important engineering task. Cooling by liquid helium is not feasible. Instead some form of liquid-cryogen-free or conduction cooling is required. Some systems make use of locally mounted Gifford-McMahon cryocoolers (typically 1.5W/4.2K each), other suggested cooling modes involve the circulation of gaseous or supercritical helium or the use of a cryogenic oscillating heat pipes.

The choice of superconductor for gantry-magnet winding is discussed in detail. Numerous proton magnets have been wound with NbTi wires – individual 0.8-0.9 mm diameter wires, a (6 + 1)-wire twisted cable, parallel stacks of 1.6 mm square insulated wires. A high-temperature-superconducting (HTS) magnet (bore field 1.2 T), conventionally wound with REBCO tape (4 mm x 0.1 mm), has been designed built and tested. In general the bore fields, even of SC magnets, have been relatively low, often less than 3 T. But for future carbon-ion systems, especially when gantry size and hence dipole bend radius is to be minimized, bore fields of order 6 T will be needed. To satisfy this requirement in a cryogen-free magnet superconductors with critical temperatures and critical fields higher than those of NbTi will be needed. A review of the properties of several low-temperature- and high temperature superconductors indicates that REBCO is the material of choice – not in the form of a difficult-to-wind tape but as a wire (or cable) made from narrow tapes spirally wrapped along a thin wire core.

Finally, an important “Prospect for Improvement” would be the introduction into the system of MRI image guidance. In order to take advantage of the Bragg peak effect the exact targeting of the tumor and positioning of the patient must figure into the treatment. The charged-particle beam has to be guided by image visualization using X-ray, CT, and hopefully MRI. An error in the target depth of a few mm that may result in only a few percent change in photon dose may lead to a 100% change in the proton dose (67). Unlike MRI-guided photon therapy the direct interaction of the magnetic field with the charged particle beam presents a huge challenge such that MRI image-guided proton/particle therapy has not yet been available in clinical practice. Modeling studies have been undertaken on the general topic of beam-line/magnetic field interaction using, for example, the software GEANT4 (GEometry And Tracking) a platform for simulating the passage of charged particles through matter using a Monte Carlo method. The paper concludes by noting that a comprehensive design of accelerators, gantries, magnets and imaging systems for particle beam therapy would be the best way to produce the most efficient and cost effective particle-beam therapy system.

## Author Contributions

EC wrote the first draft of the manuscript. LL and NG wrote sections of the manuscript. All authors contributed to manuscript revision, read, and approved the submitted version.

## Funding

This work was supported by the National Institute of Biomedical Imaging and Bioengineering, under grant R01EB018363.

## Conflict of Interest

The authors declare that the research was conducted in the absence of any commercial or financial relationships that could be construed as a potential conflict of interest.

## Publisher’s Note

All claims expressed in this article are solely those of the authors and do not necessarily represent those of their affiliated organizations, or those of the publisher, the editors and the reviewers. Any product that may be evaluated in this article, or claim that may be made by its manufacturer, is not guaranteed or endorsed by the publisher.

## APPENDIX A: Magnetic Rigidities of Proton- and Carbon-Ion Beams

### A1: Magnetic Rigidity of a 250 MeV Proton Beam

Velocity of light *c* = 2.998 x 10^8^ m/s
Rest mass of proton *m_o_* = 1.6712 x 10^-27^ kg
Charge on proton *q* = 1.602 x 10^-19^ coulomb (s.A)
Beam kinetic energy *E* = 250 MeV = 4.006 x 10^-11^ J

(A1)
$$\begin{array}{ll} & E=m_{o}c^{2}(\gamma -1)\\ hence & \gamma =E/(m_{o}c^{2})+1=1.2667 \end{array}$$

(A2)
$$\begin{array}{ll} & \gamma =1/\surd [1-{\left(v/c\right)}^{2}]\\ hence & v=c\surd [1-1/\gamma^{2}]=1.8402\;\times \;10^{8}\;m/s \end{array}$$

**Magnetic rigidity, *R*** = *m_o_γv/q* = 2.432 (kg/s^2^.A).m or T.m

### A2: Magnetic Rigidity of a 430 MeV/u (5.16 GeV) Carbon-Ion Beam

Rest mass of carbon ion *m_o_* = 1.994 x 10^-26^ kg
Charge on 6+ carbon ion *q* = 6 x 1.602 x 10^-19^ coulomb (s.A)
Beam kinetic energy *E* = 5.16 GeV = 8.2684 x 10^-10^ J

(A3)
$$\begin{array}{ll} & E=m_{o}c^{2}(\gamma -1)\\ hence & \gamma =E/(m_{o}c^{2})+1=1.461353 \end{array}$$

(A4)
$$\begin{array}{ll} & \gamma =1/\surd [1-{\left(v/c\right)}^{2}]\\ hence & v=c\surd [1-1/\gamma^{2}]=2.18615\;\times \;10^{8}\;m/s \end{array}$$

**Magnetic rigidity, *R*** = *m_o_γv/q* = 6.6275 (kg/s^2^.A).m or T.m

## APPENDIX B: Some Gantries with Normal-Conducting Magnets

### B1: Varian Medical Systems, Inc., Palo Alto, CA, USA

Ref (70).

Varian ProBeam^®^ technology is implemented in Gantry 3 of the PROSCAN facility of the Paul Scherrer Institute’s (PSI) Center for Proton Therapy (CPT), Villigen, Switzerland (41). The gantry consists of a pair of bending dipoles (45° and 135°), 5 quadrupoles, and 3 orbit correction magnets. A degrader adjusts the proton beam-energy from 70 MeV to 230 MeV and the fields of the magnets downstream change in synchronism. Varian ProBeam^®^ technology is also being implemented at:

➢ The Maryland Proton Treatment Center (MPTC), University of Maryland BioPark, West Baltimore, MD, USA
➢ Scripps Proton Therapy Center, San Diego, CA, USA
➢ The Rinecker Proton Therapy Center, Munich, Germany

Implementation is also pending at 13 other sites worldwide.

### B2: Hitachi, Ltd, Tokyo, Japan

Ref (42).

Hitachi’s system consist of three bending magnets (BM160° upwards, BM2 60° downwards and BM3, 90° downwards) and six quadrupole magnets (5). The gantry length is 6.9 m and the rotating diameter is 10.0 m. Correction (or steering) magnets compensate for the results of gantry frame distortion during rotation. A beam- energy range of 70 MeV to 250 MeV provides magnetic rigidities, *R*, of 1.23 to 2.43 Tm. A maximum magnetic field, *B*, of 1.6 T calls for a dipole bend radius *ρ = R/B* = 1.52 m. Hitachi systems (including “PROBEAT-V”) have been installed at:

➢ The Proton Medical Research Center at the University of Tsukuba Hospital, Tsukuba, Japan
➢ The M.D. Anderson Proton Therapy Center, M.D. Anderson Cancer Center (MDACC), University of Texas, Houston, TX, USA
➢ The Mayo Clinic Proton Beam Therapy Program, Rochester, MI, USA
➢ The Mayo Clinic Proton Beam Therapy Program, Phoenix, AZ, USA
➢ St. Jude Red Frog Events Proton Therapy Center, Memphis, TN, USA.

### B3: Ion Beam Applications (IBA) Louvain-la-Neuve, Belgium

In IBA’s Proteus^®^ 235 system a beam of energy 230 MeV extracted from a normal-conducting isochronous cyclotron was adjusted to 60-230 MeV by a graphite degrader and energy selection system. In 2010 IBA claimed that system to be the most commercially successful so far (7). As reported in (7), systems operating in 2010 in the US were located at:

➢ The Francis H. Burr Proton Therapy Center, Massachusetts General Hospital, Boston
➢ Midwest Proton Therapy Institute, Bloomington, IN (gantry only)
➢ University of Florida Proton Therapy Institute, Jacksonville, FL
➢ Roberts Proton Therapy Center, University of Pennsylvania Health System, Philadelphia, PA
➢ Procure Proton Therapy Center, Oklahoma City, OK
➢ Hampton University Proton Therapy Institute, Hampton, VA

and elsewhere at:

➢ Wanjie Proton Therapy Center, Zibo, China
➢ National Cancer Center, Ilsan, Korea
➢ Centre de Protonthérapie de l’Institut Curie, Orsay, France

Many others were under construction or installation.

The IBA Proteus^®^ 235 was succeeded by the IBA ProteusONE^®^ system with its 230 MeV superconducting synchrocyclotron S2C2 (**Table 5**). Nevertheless beam handling in ProteusONE^®^ was still achieved with normal-conducting magnets. As reported in (71) IBA’s compact gantry has a diameter of only 7.2 m. It is furnished with 3 dipole bending magnets, 7 quadrupoles, and 4 steering magnets. The beam energy of 230 MeV dictates a magnetic rigidity *R* = 2.324 Tm (**Table 7**); a maximum field, *B*, of 1.41 T calls for a bending radius *ρ = R/B* = 1.65 m. ProteusONE^®^ systems have been delivered to:

➢ The Rutherford Cancer Centre, South Wales, UK
➢ Cyclhad (Cyclotron for Hadron Therapy), Caen, France
➢ Hokkaido Ohno Memorial Hospital, Sapporo, Japan
